# Pathways of Protein Secretion in Prokaryotes and Eukaryotes: Molecular Mechanisms, Biological Functions, and Therapeutic Opportunities

**DOI:** 10.1002/mco2.70798

**Published:** 2026-06-07

**Authors:** Qiyuan Yang, Jingfei Shi, Linlin Xu, Guangrui Zhao, Yue Chen, Lin Cheng, Xiaokang Li, Zhigang Sun, Shuhong Huang

**Affiliations:** ^1^ School of Clinical and Basic Medicine Shandong Provincial Hospital Affiliated to Shandong First Medical University Jinan Shandong China; ^2^ Department of Dermatology Shandong Provincial Hospital Affiliated to Shandong First Medical University Jinan Shandong China; ^3^ Department of Thoracic Surgery Central Hospital Affiliated to Shandong First Medical University Jinan China

**Keywords:** bacterial secretion systems, extracellular vesicles, Golgi bypass, secretory autophagy, targeted therapy, unconventional protein secretion (UcPS)

## Abstract

Protein secretion is a fundamental biological process essential for cellular communication, matrix remodeling, and overall homeostasis across all domains of life. While the classical endoplasmic reticulum (ER)‐to‐Golgi pathway has long been recognized as the primary route for eukaryotic protein export, decades of research reveal that numerous cytosolic and membrane proteins bypass this canonical route. This alternative paradigm, termed unconventional protein secretion (UcPS), encompasses direct transmembrane translocation, vesicle‐mediated release, and Golgi‐bypass mechanisms. Simultaneously, prokaryotes utilize highly specialized secretion machineries to deliver effector proteins. Despite these established frameworks, the precise molecular regulation, cargo sorting mechanisms, and dynamic crosstalk between these diverse pathways remain incompletely understood. Here, we comprehensively review the molecular mechanisms of UcPS and prokaryotic secretion systems, synthesizing their evolutionary adaptations and operational frameworks. By mapping nonvesicular pores, extracellular vesicles intermediates, and complex bacterial nanomachine assemblies, we delineate how cells rapidly mobilize proteins under stress. Furthermore, we highlight the dual role of these pathways in driving physiological adaptation versus fueling pathological dissemination. Abnormalities in these secretory nodes are now recognized as primary drivers of neurodegeneration, inflammatory disorders, and cancer metastasis. Consequently, manipulating UcPS mechanisms offers promising, multidimensional therapeutic opportunities for future medicine.

## Graphical abstract

This graphical abstract delineates noncanonical protein secretion systems across eukaryotes and prokaryotes. Eukaryotic UcPS covers four ER–Golgi bypass pathways, enabling rapid leaderless protein export via direct transmembrane translocation or vesicle‐mediated release. Prokaryotic specialized secretion systems (T3SS, T6SS, T7SS) span the bacterial envelope to deliver effectors in one step. Our review systematically deciphers their molecular mechanisms, physiological and pathological roles, as well as translational therapeutic potential.

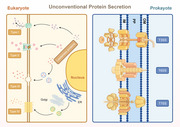

## 1 Introduction

Protein secretion is a fundamental biological process essential for cellular homeostasis across all domains of life. In prokaryotes, the general secretion (Sec) and twin‐arginine translocation (Tat) pathways serve as the primary, evolutionarily conserved systems for transporting nascent or fully folded proteins across the cytoplasmic membrane. Whereas the classical protein secretion pathway has long been established as the primary route for protein export in eukaryotic cells. This canonical process begins with protein synthesis on the ER, relies on sequential vesicular transport to the Golgi apparatus for processing, and culminates in Soluble NSF Attachment Protein Receptor (SNARE)‐mediated fusion with the plasma membrane [1]. However, decades of research have revealed that not all proteins adhere to these conventional routes. Pioneering studies in the 1990s on key proteins, such as IL‐1β, HIV‐Tat, and CFTR, established that certain factors can be secreted independently of the Golgi apparatus [2, 3, 4], particularly under stress or pathological conditions, prompting the conceptualization of UcPS.

Historically regarded as a minor alternative, UcPS has rapidly evolved into a focal point of modern cell biology due to its critical roles in inflammation, neurodegeneration, and cancer. Currently, eukaryotic UcPS is systematically classified into four distinct types [5]. Type I and Type II involve the direct transmembrane transport of cytosolic proteins lacking signal peptides, whereas Type III relies on complex vesicular carriers such as exosomes, microvesicles (MVs), and autophagosomes. Type IV encompasses proteins that successfully enter the ER but bypass Golgi maturation. Concurrently, studies into prokaryotic secretion have uncovered diverse and highly specialized machineries, ranging from the fundamental Sec and Tat pathways to the intricate Type I through Type XI secretion systems (T1SS–T11SS), which mediate the targeted delivery of effector proteins [6]. Furthermore, recent advancements have expanded the scope of cellular transport to include emerging structures like migrasomes, which facilitate spatially localized intercellular communication during cell migration [7].

Despite the establishment of this comprehensive classification framework, the precise molecular regulation, cargo sorting mechanisms, and dynamic crosstalk between these diverse pathways remain incompletely understood. Furthermore, the growing association of UcPS with severe human pathologies—ranging from neurodegenerative disorders to cancer—has deeply underscored both its pathological significance and its immense therapeutic potential. Because UcPS plays a dual role in driving physiological cellular adaptation and fueling pathological dissemination, intervening at these critical Sec nodes has emerged as a promising frontier in precision medicine. Therefore, this review aims to synthesize recent mechanistic insights into UcPS, delineate its multifaceted roles in disease progression, and investigate novel, targeted intervention strategies. In this article, we begin by elucidating the distinct molecular mechanisms underpinning UcPS, detailing nonvesicle‐mediated direct transmembrane transport (Types I and II), vesicle‐mediated pathways encompassing exosomes, MVs, and autophagosomes (Type III), and Golgi bypass routes (Type IV). We subsequently explore the structural architecture, pathogenicity, and effector delivery strategies of prokaryotic‐specific secretion mechanisms, alongside emerging pathways like migrasome‐mediated transport. Next, we thoroughly discuss the biological implications of these pathways in maintaining intercellular communication and extracellular matrix (ECM) remodeling, followed by their pathological involvement in neurodegenerative diseases, cancer, and immune evasion. Finally, we critically assess the current therapeutic opportunities that target these unique secretory mechanisms and outline pressing future research directions necessary for bridging basic science with clinical relevance.

## 2 Classical Secretion Pathways

### 2.1 Sec Pathway

The Sec pathway is the most fundamental and conserved protein transmembrane transport system shared by prokaryotes and eukaryotes. In eukaryotes, its main function is to transport nascent proteins into the ER, thus entering the subsequent Sec pathway. In prokaryotic cells, however, it is the major pathway across the cytoplasmic membrane [8].

The Sec pathway is the first identified pathway for bacterial proteins to be secreted across the cytoplasmic membrane to the periplasm and outer membrane (OM). Its core components include the SecYEG transmembrane channel, the SecA ATPase motor, and a variety of molecular chaperones, such as SecB and signal recognition particle (SRP). Preproteins are recognized and targeted via their N‐terminal signal peptides, which act as molecular tags to direct proteins to the Sec translocation system. Among them, signal peptides with high hydrophobicity are recognized by the SRP [9, 10], which then binds to its receptor FtsY to form the ribosome–nascent chain complex–SRP–FtsY complex. This complex is targeted to the SecYEG channel for cotranslational translocation, a process that is primarily applicable to membrane proteins [11]. In contrast, signal peptides with low hydrophobicity escape the recognition and surveillance of SRP and instead bind to trigger factor (TF) or SecA [12, 13]. Preproteins are primarily bound by the molecular chaperone SecB or TF to maintain their unfolded translocation‐competent state [14, 15, 16, 17], and are subsequently delivered to either cytoplasmically free SecA or SecA that is already associated with the SecYEG channel to initiate posttranslational translocation [18, 19], a pathway predominantly dedicated to Sec proteins. Cytoplasmic SecA dimers first anchor to the SecYEG channel on the plasma membrane via a single protomer, assembling into the SecYEG–SecA translocase holoenzyme [19, 20]. After preproteins are delivered to this complex, SecA recognizes the signal peptides and mature domains of preproteins through two distinct binding sites, respectively [21, 22]. Binding of the signal peptide induces conformational relaxation and elongation of the SecA dimer [23], and this allosteric effect is transmitted to SecYEG, potently activating the ATPase activity of SecA [21, 24]. Following ATP binding to SecA, the association between SecA and SecYEG is further tightened [25], and its IRA1 two‐helix finger domain inserts into the cytoplasmic funnel of SecY [26, 27, 28], accompanied by the coinsertion of a ∼20–30 amino acid residue preprotein segment into the channel pore [29]. ATP binding and hydrolysis drive cyclic conformational changes in SecA, which propel the stepwise translocation of preproteins across the SecYEG channel via the power stroke mechanism; among these processes, ATP hydrolysis stimulated by preproteins induces SecA monomerization [21, 23, 30, 31]. Once most preproteins accomplish transmembrane translocation, SecA releases ADP and dissociates from SecYEG, then redimerizes in the cytoplasm and undergoes conformational resetting to enter the next round of the translocation cycle [23, 25]. Meanwhile, the SecYEG channel possesses an intrinsic dynamic regulatory capacity: its pore ring is composed of six hydrophobic amino acid residues [32], which restricts the channel pore size to an extremely narrow aperture in the resting state. Upon preprotein entry, the pore ring dilates to form a sealing gasket around the polypeptide chain and permit its passage [33]. The plug structure seals the periplasmic funnel in the idle state of the channel to prevent ion leakage [34], and undergoes translocation upon preprotein entry, thereby inducing channel opening [33]. For membrane proteins, the lateral gate of SecYEG is jointly formed by transmembrane helices 2, 3 and 7, 8 of SecY [35]. This gate can open to permit the lateral insertion of hydrophobic transmembrane helices into the lipid bilayer. Upon completion of translocation, the signal peptide is cleaved and removed by the membrane‐anchored signal peptidase (SPase) at the conserved AXA cleavage motif, and the mature protein is released into the periplasmic space to undergo proper folding with the assistance of periplasmic chaperones [36, 37].

Compared with the prokaryotic Sec pathway, the eukaryotic Sec pathway is highly conserved in core mechanisms yet characterized by a more sophisticated system: its core channel is the Sec61αβγ complex, which is homologous to the prokaryotic SecYEG and localized to the ER membrane rather than the cytoplasmic membrane. The eukaryotic Sec pathway is dominated by the mode of cotranslational translocation, a process mediated by the cytosolic SRP [38]. As soon as the signal sequence of a nascent polypeptide chain is synthesized and exposed from the ribosome, it is recognized and bound by SRP. Subsequently, SRP targets the ribosome‐polypeptide complex carrying the nascent polypeptide chain to the SRP receptor on the ER membrane. Upon the completion of docking, the ribosome directly associates with the Sec61 channel on the ER membrane, leading to the synchronous occurrence of protein translation and transmembrane translocation, and the nascent polypeptide chain is translocated into the ER lumen in a coupled translation–translocation manner. In the meantime, subsequent studies on Saccharomyces cerevisiae have verified that the eukaryotic Sec pathway also exists in a posttranslational translocation mode [39]. The choice of translocation pathway for a protein is determined by the characteristics of its signal sequence: generally, proteins undergoing posttranslational translocation have signal sequences with lower hydrophobicity than those of proteins dependent on the SRP‐mediated cotranslational pathway. In addition, eukaryotic posttranslational translocation is independent of the SecB–SecA system that acts as the core of the prokaryotic posttranslational pathway, which forms a striking contrast to the prokaryotic mode, where most secreted proteins adopt posttranslational translocation. However, the core components and functional mechanisms of the SRP‐mediated cotranslational pathway exhibit significant evolutionary conservation between eukaryotes and prokaryotes [8].

### 2.2 Tat Pathway

The Tat system is evolutionarily conserved across prokaryotes and plant organelles and is present in the vast majority of bacteria, archaea, and the thylakoid membranes of plants and cyanobacteria. With the exception of homoscleromorph sponges, the Tat system has been completely lost from animal mitochondria [40, 41, 42, 43]. Unlike the Sec system, the Tat pathway primarily transports proteins that are fully folded in the cytosol and is strictly a posttranslational pathway [44, 45]. The assembly and translocation cycle of the Tat translocon is a dynamic, “on‐demand” process [46, 47, 48, 49]. In its resting state, the Tat receptor complex is composed of TatA, TatB, and TatC at a 1:1:1 stoichiometry [50, 51, 52]. TatB forms extensive, tight interactions with the full length of transmembrane helix 5 (TM5) of TatC, as well as the periplasmic apex of TM6, while TatA is bound to the TM6 site of TatC at this stage [50]. A conserved surface patch on the cytoplasmic face of TatC serves as the core recognition site for the twin‐arginine motif [49, 53]. Following the cytosolic synthesis of a folded Tat substrate bearing an N‐terminal Tat signal peptide containing the twin‐arginine motif, the twin‐arginine motif of the signal peptide is specifically recognized by the conserved cytoplasmic surface patch of TatC, triggering deep insertion and binding of the signal peptide into the core of the receptor complex [49, 54, 55, 56]. Subsequently, the TatBC complex undergoes a critical conformational rearrangement, in which TatC protomers switch from the head‐to‐tail arrangement in the resting state to a tail‐to‐tail conformation. This conformational change exposes the previously occupied polar cluster binding sites within its transmembrane helical region, allowing TatA to access the vacated polar cluster sites [53], which in turn drives the recruitment and oligomerization of additional TatA molecules. At this stage, the hairpin conformation of the signal peptide undergoes unhinging [55], allowing the folded substrate protein to complete transmembrane translocation via the TatA oligomer‐mediated pathway [57, 58, 59, 60]. Upon completion of translocation, the TatA oligomer dissociates, and the translocation system undergoes conformational rearrangement to revert to its resting state.

The Tat and Sec systems invariably coexist in prokaryotes and plant plastids, which poses a central challenge: how to accurately sort substrate proteins to their cognate transport pathways. The nearly invariant twin‐arginine motif of Tat signal peptides (centered on a pair of consecutive arginine residues, most often embedded within the extended consensus motif S‐R‐R‐x‐F‐L‐K) is the core determinant for specific substrate recognition by the Tat system [49, 53]. This motif occurs at an extremely low frequency in *Escherichia coli* Sec signal peptides, yet does not itself confer avoidance of recognition by the Sec system [61, 62]. In addition to the specific interaction between the twin‐arginine motif and the Tat receptor complex, two key features of Tat signal peptides serve as the true determinants for ensuring the fidelity of Tat substrate sorting. First, the c‐region of the Tat signal peptide frequently contains at least one basic amino acid residue, which acts as a “Sec‐avoidance motif” to reduce functional engagement with the Sec pathway, while having no impact on the intrinsic translocation efficiency of the Tat pathway [63, 64, 65, 66]. Second, the h‐region of Tat signal peptides is generally less hydrophobic than that of Sec signal peptides, which likely contributes to evasion of recognition by the SRP and SecA [63]. Meanwhile, the Tat and Sec pathways are not completely independent of one another; instead, they operate with a precisely coordinated division of labor, namely, a stepwise assembly mechanism for dual‐targeted proteins. The canonical example of this crosstalk is the Rieske iron‐sulfur protein from Actinobacteria: the preceding transmembrane helices of this protein are cotranslationally inserted into the membrane via the Sec pathway, while the final transmembrane helix shares the characteristics of a canonical Tat signal sequence [67, 68, 69]. Once the iron–sulfur cluster‐containing domain is fully folded and cofactor‐assembled in the cytosol, the membrane‐tethered Tat signal is recognized by the Tat system to complete the final topological assembly and subcellular localization of the protein [70]. This functional complementarity and coordinated division of labor enable bacteria to efficiently handle proteins with distinct folding states: the Sec pathway mediates co‐ and post‐translational translocation of unfolded polypeptide chains, while the Tat pathway ensures precise transmembrane transport of fully folded, cofactor‐bound proteins (e.g., key components of the respiratory chain) preassembled in the cytosol. Against the backdrop of the inherent functional overlap between the two pathways, this arrangement minimizes substrate mis‐sorting and enables precise regulation of cell membrane and membrane protein biogenesis.

### 2.3 ABC Transporters

The ATP‐binding cassette (ABC) transporter is one of the largest protein superfamilies and is present in all organisms. These transporters utilize the energy generated from ATP binding and hydrolysis to transport various substrates. Although some ABC transporters are primarily known for their roles in drug resistance and lipid transport, they also play important roles in the UcPS process in yeast and parasites, as well as in the Type I secretion systems (T1SS) of bacteria. This section outlines the structural and functional principles of ABC transporters and their emerging roles in UcPS. The human genome contains 48 coding genes, which belong to seven subfamilies. Of these, five subfamilies (A, B, C, D, and G) are involved in transmembrane transport and encode a total of 44 transporters, which exhibit broad substrate specificity and functional diversity [71]. The proteins of this family are highly modular in structure. The basic unit consists of two symmetric domains that form a half‐transporter. These half‐transporters can assemble into functional complexes through homo‐ or heterodimerization or fuse during evolution to form a complete transporter of a single‐chain polypeptide. This structural flexibility enables them to adapt to diverse physiological needs, reflecting an evolutionary trend toward increased functional versatility and complexity [72]. Each half‐transporter contains a nucleotide‐binding domain (NBD) and a transmembrane domain (TMD). The NBD contains the Walker A/B motifs and the ABC‐characteristic C‐loop. The first two mark the nucleotide‐binding site, and the latter, as a family‐specific sequence, plays a key role in ATP binding and catalysis. The TMD facilitates the directional transport of substrates, which may move toward or away from the NBDs. The energy for transport is derived from the ATP hydrolysis process in the NBD [73].

**FIGURE 1:**
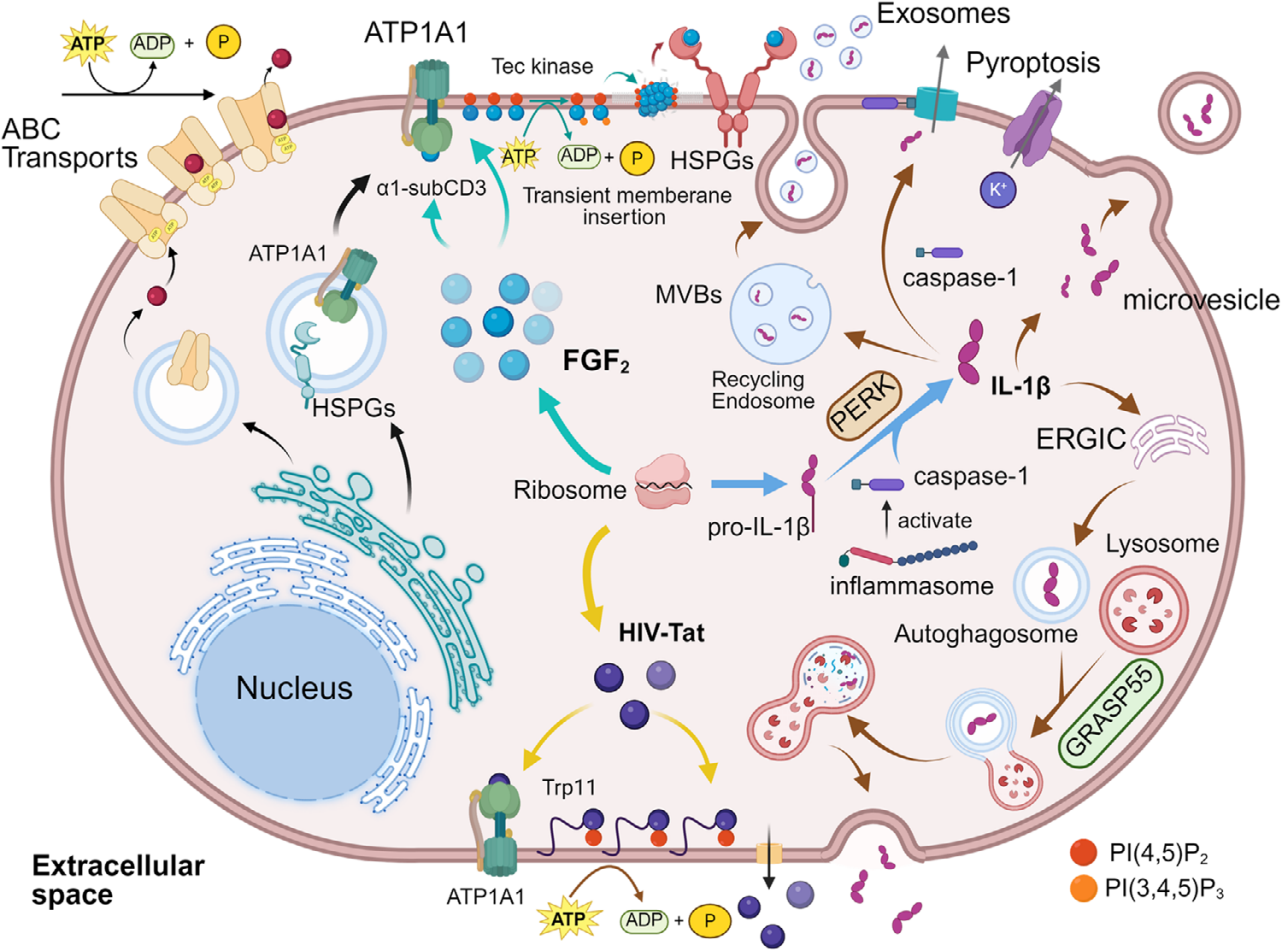
Overview of major unconventional secretion mechanisms for leaderless proteins. IL‑1β is released via microvesicles/exosomes, gasdermin D pores, or GRASP55‑mediated autolysosomes. FGF2 is recruited by PI(3,4,5)P_3_ to the plasma membrane, oligomerizes via PI(4,5)P_2_ and ATP1A1, assisted by Tec kinase, and is captured by extracellular HSPGs. HIV‑Tat binds PI(4,5)P_2_ and ATP1A1, with Trp11 inserting into the lipid bilayer. ABC transporters use ATP‑driven conformational changes to export diverse substrates. (Image created in BioRender. Shi, J. (2025) https://BioRender.com/pi0fkal.)

In substrate transport, conformational changes are at the heart of the driving mechanism: initially, the NBD binds to ATP and forms a closed dimer, inducing a conformational change in the TMD to expose the substrate binding site. Sequence variation in the TMD determines substrate specificity; substrate binding triggers ATP hydrolysis, with the released energy prompting a second conformational switch in the TMD‐efflux transporters, which move substrates from inside the cell to outside, whereas uptake transporters do the opposite. Concurrently, the NBD shifts from a closed to an open state; upon completion of transport, the now‐empty binding site repositions along with TMD back to its original side (usually the cytoplasmic side), and the NBD returns to its open state, ready for new ATP binding to initiate another cycle. This ATP‐dependent dynamic conformational cycle allows ABC transporters to efficiently accomplish transmembrane substance transport, and their structural and functional adaptability underpins the significant role this family plays in cellular material metabolism and homeostasis maintenance (Figure 1) [72]. Although most ABC transporters are involved in lipid metabolism and drug resistance‐related pathways [72], some may participate in UcPS. A considerable portion of these proteins originates not from humans but from other microorganisms. As early as 1989, it was discovered that the a‐factor mating pheromone of Saccharomyces cerevisiae is secreted extracellularly via the ABC transporter Ste6p [74], followed by findings that the m‐factor of Schizosaccharomyces pombe [75] and the hydrophilic acylated surface protein B (HASPB) of Leishmania species are also transported through ABC transporters [76, 77], though the specific mechanisms remain unclear.

## 3 UcPS in Eukaryotes

Eukaryotic UcPS comprises several mechanistically distinct routes that together allow both leaderless and conventionally targeted proteins to reach the cell surface independently of the ER–Golgi cascade. As outlined schematically in the introduction, these include direct passage across the plasma membrane, export via diverse vesicular intermediates, and Golgi‐bypass trafficking of ER‐entered cargos. In the following subsections, we first examine nonvesicle‐mediated pathways, in which protein–lipid interactions and stress‐induced pores in the plasma membrane create transient conduits for secretion, before turning to vesicle‐dependent and Golgi‐bypass mechanisms.

### 3.1 Nonvesicle‐Mediated UcPS

Nonvesicle‐mediated UcPS mainly involves Type I UcPS. Type I UcPS denotes a direct transmembrane transport mechanism that enables rapid protein release independent of the classical ER–Golgi pathway. This process is characterized by charge‐dependent interactions between proteins and specific phospholipids in the plasma membrane, which drive the oligomerization of secretory proteins at the membrane surface and the formation of transmembrane pores [78]. The mechanisms driving pore formation are broadly categorized into two types: “self‐made” lipidic pores and inflammation‐driven heterologous pore formation [79]. During this process, conformational rearrangements of proteins and perturbations of the lipid bilayer work in concert to allow monomers or oligomers of secretory proteins to pass through transiently formed pores and be released extracellularly [80]. This method can achieve rapid protein release even in the absence of vesicular transport, particularly catering to the needs of cells for immediate responses to microenvironmental changes under pathological or stress conditions.

#### 3.1.1 Self‐Made Lipidic Pores

Fibroblast growth factor 2 (FGF2) is a mitogen secreted by various cells during development. It plays crucial roles not only in physiological processes such as angiogenesis but also in pathologies that promote tumor progression, implicating it in the malignancy of both solid and hematological cancers [78]. FGF2 is now established to be secreted unconventionally via Type I UcPS, a pathway defined by its direct translocation across the plasma membrane. This process enables the direct formation of pores in the plasma membrane to facilitate transmembrane transport in the absence of external stimuli. This translocation involves three key steps: (1) PI(4,5)P_2_‐mediated recruitment of FGF2 to the inner leaflet; (2) PI(4,5)P_2_‐induced oligomerization and membrane insertion, leading to pore formation; and (3) capture of FGF2 at the outer leaflet by membrane‐proximal heparan sulfate proteoglycans (HSPGs) (Figure 1). Collectively, these steps enable the efficient translocation of FGF2 from the intracellular to the extracellular space at a rate several orders of magnitude faster than the conventional ER–Golgi‐dependent pathway [81].

During cell signaling, the Na^+^/K^+^ ATPase α1 subunit serves as the first contact site between FGF2 and the inner leaflet of the plasma membrane, providing a platform for FGF2 anchoring. Specifically, FGF2 associates with the cytoplasmic domain (α1‐subCD3) of the Na^+^/K^+^‐ATPase α1 subunit, an interaction that involves the second cysteine residue (C77) on the FGF2 surface [82]. This binding induces conformational remodeling in FGF2, which is critical for subsequently exposing its PI(4,5)P_2_‐binding site [80]. The presence of Lys54 and Lys60 on the FGF2 surface is also essential for this initial membrane contact. Following this recruitment, FGF2 binds to PI(4,5)P_2_ in the inner leaflet via electrostatic interactions with a unique cluster of basic amino acids (K127, R128, K133), thereby achieving precise membrane localization. This dynamic interplay not only drives the targeted recruitment of FGF2 but also guides its ordered arrangement on the membrane surface via spatial constraints [83], ultimately leading to oligomerization and membrane pore formation in a PI(4,5)P_2_‐dependent manner.

It has been revealed that the PI(4,5)P_2_‐binding pocket of a single FGF2 subunit is localized to the periphery of the oligomer during oligomer assembly to achieve complementary binding to the polar head group of PI(4,5)P_2_ [84]. This precise spatial arrangement mechanism constitutes the core molecular basis for the transmembrane transport of FGF2. The process of FGF2 membrane remodeling begins with the C95–C95 disulfide bond‐mediated dimerization of FGF2 on the membrane surface, generating building blocks for higher‐order FGF2 oligomers that drive the formation of membrane pores. As the oligomer hierarchy increases, its affinity for PI(4,5)P_2_ gradually strengthens. Under this synergistic effect, FGF2 oligomers insert into the membrane, disturbing the arrangement of the lipid bilayer through hydrophobic interactions, leading to changes in local membrane curvature. Eventually, a circular transmembrane channel is constructed within the lipid bilayer [85]. This nanoscale pore not only provides a specific insertion site for the FGF2 complex but also enables transmembrane translocation of the molecule by establishing a hydrophilic microenvironment. Cholesterol enhances its accessibility to FGF2 binding by promoting the formation of PI(4,5)P_2_ clusters and alters membrane properties to facilitate FGF2 translocation processes [80]. In addition, this process is also regulated by Tec kinase. It is recruited to the inner leaflet of the plasma membrane through interaction with the phosphoinositide phosphatidylinositol (3,4,5)‐trisphosphate [PI(3,4,5)P_3_]. Once activated, it forms a heterodimeric complex with FGF2, leading to the phosphorylation of FGF2 at tyrosine 82. This phosphorylation is crucial for the efficiency of FGF2 oligomer membrane insertion and the effective secretion of FGF2 by cells [86].

The model of “transmembrane FGF2 oligomers” is still at the speculative stage and lacks direct experimental evidence. However, regardless of its true mode, this process is essentially diffusion controlled. Thus, specific mechanisms are required to ensure the effective net transfer of FGF2 molecules from the intracellular to the extracellular space [87]. Existing studies suggest that this process is largely dependent on extracellular traps made up of cell surface HSPGs connected to glypican‐1 [88]. These sites are capable of capturing and enriching FGF2 that is secreted near the cell surface. They form a ternary complex consisting of extracellular FGF2, HSPGs, and FGFRs, which mediates FGF2 signaling and protects FGF2 on the cell surface from degradation [89]. Notably, although FGFRs play a crucial role in the signaling process, they are not essential components for promoting the secretion of FGF2 [90]. Moreover, FGF2 can also spread between cells through direct cell‐to‐cell contact, which may involve the direct exchange of heparan sulfate chains that are physically associated with the surfaces of adjacent cells. This process not only demonstrates the role of FGF2 in intercellular communication but also suggests its potential unique transport pathways at the tissue level [91].

The secretion mechanism described for FGF2 is shared by other proteins, such as HIV‐Tat. HIV‐Tat is also translocated directly across the plasma membrane in a process dependent on PI(4,5)P_2_ at the inner leaflet and HSPGs at the outer leaflet. Unlike FGF2, HIV‐Tat requires a lipid membrane to complete its binding with PI(4,5)P_2_, and this binding is reinforced by the hydrophobic insertion of Tat's Trp11 into the inner membrane (IM) leaflet [92]. The membrane pores formed by HIV‐Tat are interpreted as an intermediate state of Tat membrane translocation, and oligomerized HIV‐Tat is more efficient in forming these pores. Specifically, significant membrane activity is exhibited at a protein concentration as low as 0.1 µM for HIV‐Tat, while at least 1 µM of protein concentration is required for FGF2 to induce the formation of a large number of membrane pores. Furthermore, unlike FGF2, HIV‐Tat oligomerization and pore formation occur independently of intermolecular disulfide bonds. Instead, the protein undergoes a conformational change from an intrinsically disordered state to a membrane‐inserted oligomer, engaging the hydrophobic membrane core via its central hydrophobic domain [93]. Eventually, the Tat protein may insert into the membrane and form temporary pores or channels, allowing itself to pass through.

#### 3.1.2 Inflammation‐Driven Heterologous Pore Formation

Inflammation can trigger the formation of heterologous pores in the plasma membrane, facilitating the large‐scale, rapid release of cytokines such as IL‐1β from macrophages into the extracellular space (Figure 1).

IL‐1β is a potent proinflammatory cytokine produced and secreted by various cell types, such as monocytes and macrophages [94]. Due to its widespread distribution among target cells, it plays a role in numerous inflammation‐inducing and immune‐regulating processes. In the initial stage, IL‐1β is synthesized intracellularly in the form of a 31‐kDa inactive precursor (pro‐IL‐1β), and its transcriptional expression is triggered by pathogen‐associated molecular patterns (PAMPs). When PAMPs are recognized by pattern‐recognition receptors (PRRs) on the surface of macrophages, they activate the downstream gene regulatory network, thus initiating the immune response [95]. The processing and maturation of pro‐IL‐1β, however, require a second signal mediated by the inflammasome. Inflammasome activation is typically triggered by damage‐associated molecular patterns (DAMPs) released by tissue damage or stress as a key factor. The recognition of DAMPs promotes the assembly of the inflammasome complex, which is formed by the homotypic interaction of apoptosis‐associated speck‐like protein containing a CARD (ASC), cytoplasmic PRRs (such as NOD‐like receptors), and procaspase‐1. After the inflammasome is assembled, procaspase‐1 is recruited through its homotypic interaction with ASC via its CARD domain, facilitating the activation of caspase‐1. And after caspase‐1‐dependent processing of pro‐IL‐1β, mIL‐1β is rapidly secreted from the cells [96]. A critical and long‐standing question, however, concerns the mechanism by which mature IL‐1β is secreted, as it lacks a signal peptide and does not enter the conventional secretion pathway. Evidence suggests that IL‐1β secretion involves multiple unconventional mechanisms rather than a single unified pathway [97]. Here, we will summarize one of them and provide a sufficient theoretical basis for the proposed multiple mechanisms.

Previous experiments have revealed that numerous factors are capable of inducing the release of IL‐1β. These include various inflammasomes (e.g., NLRP3, NLRC4, NLRP1) and cytosolic sensors such as AIM2 and RIG‐I [97]. Infections by pathogenic bacteria such as Salmonella typhimurium, Shigella flexneri, Legionella pneumophila, and Pseudomonas aeruginosa, as well as DAMPs like uric acid crystals, ATP, aluminum adjuvant, and β‐amyloid (Aβ) peptide, activate the NLRC4 or NLRP3 inflammasomes [97, 98]. In turn, this leads to the activation of caspase‐1 within macrophages. This leads to a rapid caspase‐1‐dependent cell death, which is known as pyroptosis. Pyroptosis represents a highly proinflammatory form of programmed cell death. Its characteristic feature is the mediation of the lysis of infected autophagic cells. Simultaneously, a large quantity of proinflammatory factors is released. For instance, during pyroptosis, IL‐1β experiences caspase‐1‐dependent activation and secretion [99].

Further studies have found that in this pathway, IL‐1β does not bind to PI(4.5)P_2_ and forms pores like FGF2. Instead, hyperpermeabilization of the macrophage membrane occurs during IL‐1β secretion [99, 100]. So, how does hyperpermeabilization occur? After the inflammasome is activated, conventional caspase‐1 and unconventional caspase‐4/‐5 are activated. They cleave gasdermin D (GSDMD) at D275 (FLTD|GVP in humans), separating its 31‐kDa active N‐terminal (GSDMD‐N) from its 22‐kDa self‐inhibitory C‐terminal fragment [101, 102, 103]. GSDMD‐N preferentially interacts with negatively charged membrane lipids, such as phosphatidylserine in the inner leaflet of the cell membrane and cardiolipin in the inner and outer leaflets of the bacterial membrane. During this process, it oligomerizes to form a ring‐shaped structure. This structure targets the cytoplasm, mitochondria, nucleus, and bacterial membranes, increasing the permeability of these membranes and leading to the loss of intracellular structures [104, 105, 106]. The GSDMD ring forms a pore in the plasma membrane, ultimately resulting in cell lysis and providing a direct channel for the transfer of IL‐1β to the extracellular environment.

In recent years, studies have found that phagocytes that do not undergo pyroptosis can also release IL‐1β in a state called “hyperactive” [107, 108]. Experiments have demonstrated that the formation of GSDMD in this state is basically consistent with this part of the pyroptosis pathway, and GSDMD is necessary for the release of IL‐1β by cells in this state [108]. Although the amount of IL‐1β secreted in this way is less than that secreted via the pyroptosis pathway, phagocytes will increase the IL‐1 family cytokines in their secreted factor pool, and these cells may continue to influence immunomodulatory events [108]. The reason these cells do not undergo pyroptosis may be that endosomal sorting complexes required for transport (ESCRT) can remove the GSDMD pores on the cell membrane in the form of vesicles, enabling the cells to limit pyroptosis while allowing limited GSDMD‐dependent cytokine release [108, 109, 110].

Another “pore” formed during inflammation is the purinergic receptor P2X7 (P2X7R), which is more accurately characterized as an ATP‐activated potassium channel. Past studies have demonstrated that P2X7R is the receptor mediating the effects of ATP [111, 112]. While P2X7R has been implicated in the secretion of proteins like TG2 [113], research in the context of IL‐1β indicates that it does not serve as a direct transport channel. Instead, it promotes ATP‐mediated caspase‐1 activation and pro‐IL‐1β processing by inducing potassium ion efflux. The release of IL‐1β may occur through the “two‐signal model”: the first signal is the stimulation of Toll‐like receptors, leading to the accumulation of pro‐IL‐1β in the cytoplasm; the second signal is the ATP‐dependent stimulation of P2X7R, promoting inflammasome‐mediated caspase‐1 activation [114, 115, 116]. P2X7R reduces the intracellular potassium ion concentration by inducing potassium ion efflux, triggering the assembly of the NLRP3 inflammasome complex (NLRP3–ASC–NEK7–caspase‐1), which in turn leads to caspase‐1 cleavage and the release of mIL‐1β [117]. The release of mIL‐1β can occur through passive release after cell death and secretion via modified lysosomes, exosomes, or plasma‐membrane‐derived MVs, and P2X7R is also the main driver of these pathways [117]. However, recent studies have also found that after P2X7R activation, there is also an NLRP3‐independent pathway for IL‐1β release, and in this pathway, the cleavage of pro‐IL‐1β is not caused by caspase‐1, cysteine protease, or aspartic proteases [118]. It is worth noting that there is also a synergistic effect between P2X7 and GSDMD. GSDMD, through its pore‐forming ability, enables molecules such as IL‐1β, P2X7, and caspase‐1 to enter microparticles. Subsequently, P2X7 on the surface of the microparticles binds to ATP and is activated, mediating the formation of microparticle pores and releasing IL‐1β to target cells [119].

In addition, apart from the above caspase‐1‐dependent activation pathway, there is also a noncaspase‐1‐dependent mechanism for the activation of IL‐1β. This mechanism plays a role in the inflammatory response after sterile injury or infection, manifested as a caspase‐1‐independent IL‐1β‐dependent inflammatory response. Research has confirmed that when injury causes necrosis of mouse peritoneal macrophages, IL‐1β is released only in the form of inactive pro‐IL‐1β [120]. Further experiments showed that after treatment with the calcium ionophore A23187 and the detergent saponin, which are expected to impair macrophage viability, no form of IL‐1 other than pro‐IL‐1β was detected in the extracellular environment [121]. This indicates that only immature pro‐IL‐1β is released into the extracellular space during cell necrosis. However, under pathological conditions such as sterile tissue injury [122] and inflammatory arthritis, proteases such as proteinase‐3, cathepsin G, and neutrophil elastase—all stored in neutrophil azurophilic granules—can process pro‐IL‐1β into its mature form for release. The bioactivity of this neutrophil‐derived mIL‐1β is comparable to that of its caspase‐1‐derived counterpart [123]. Therefore, this process reveals that the inactive IL‐1β released after cell necrosis can be processed by the proteases carried by neutrophils during neutrophil infiltration, thus enabling a caspase‐1‐independent IL‐1β‐dependent inflammatory response. Table 1

**TABLE 1:** Comparison of unconventional secretion pathways for IL‐1β, FGF2, and HIV‐Tat.

	IL‐1β	FGF2	HIV‐Tat
Type of secretion pathway	Direct budding, MVB‐mediated exocytosis, GSDMD pore‐mediated passive release, autophagolysosome membrane fusion [124, 125, 126, 127]	Lipid raft‐ and PI (4,5)P_2_‐dependent direct transmembrane transport [128, 129]	Pore formation for direct membrane penetration [93]
Key regulatory molecules	NLRP3 inflammasome, caspase‐1, GSDMD, NEK7 [130]	Tec kinase family, [87] Na^+^/K^+^‐ATPase, HSPGs	Na^+^/K^+^‐ATPase, Trp11 [93]
Physiological/pathological triggers	NLRP3 activation, inflammatory stimuli, pyroptosis [99]	Hypoxia, cell injury, and absence of growth factor signaling	HIV infection, host cell stress [131]
Dependency on membrane damage	Yes	No (local rearrangement)	No (transient pores)
Energy dependence	Low	High [132]	High [133]
Oligomerization	Pro‐oligomerization promotes activation of the cleavage process	Complex formation with HSPGs [89]	Complex formation with viral RNA or host proteins
Chaperones/accessory factors	HSP90 [134], ASC	S100A13 [135]	Cyclin T1 [136], host RNA‐binding proteins
Disease associations	Rheumatoid arthritis [137], Parkinson's disease [138], and autoinflammatory disorders	Tumor angiogenesis, impaired tissue repair, fibrosis, neurodegenerative diseases [139]	AIDS, cardiovascular disease, HAND, renal failure, cancer [140, 141, 142]
Potential therapeutic targets	NLRP3 inflammasome, caspase‐1	PI3K pathway, lipid raft structures	Tat protein neutralizing antibodies

*Abbreviations*: AIDS, acquired immunodeficiency syndrome; HAND, HIV‐associated neurocognitive disorders.

### 3.2 Vesicle‐Mediated UcPS

Proteins without signal peptides can be exported out of the cell via membrane transport, not only through direct transmembrane transport but also by utilizing vesicles as carriers. This process involves multiple organelles and vesicles, including autophagosomes, exosomes, MVs, lysosomes, and others. This section will explore the biogenesis, regulation, cargo selection, and release of these vesicles and elaborate on their roles in physiological communication and pathological dissemination.

#### 3.2.1 Exosome

Exosomes are tiny vesicles with diameters ranging from 30 to 150 nm that can serve as a medium for intercellular communication, and their formation originates from early endosomes (EEs). EEs are the initial sites for the fusion of endocytic vesicles and are responsible for sorting membrane proteins and lipids. Their formation depends on the activation of Rab5, effector‐mediated membrane fusion, and phosphatidylinositol‐3‐phosphate (PI3P) synthesis [143]. Subsequently, the ESCRT mediates the process of membrane invagination to form intraluminal vesicles (ILVs). ESCRT‐0, ‐I, and ‐II can work synergistically with associated protein factors to induce membrane curvature. The ESCRT‐III subunits promote membrane remodeling by assembling with Alix and other proteins and interacting with helical filaments of various types of vacuolar protein sorting (VPS) complexes, including an ATPase [144, 145, 146]. Then, when ESCRT‐III is removed, the neck of the ILVs breaks, leading to division [147, 148]. The Mon1–Ccz1 GEF complex can drive the conversion from Rab5 to Rab7, completing a crucial cascade reaction for endosome maturation [149]. Meanwhile, the ESCRT complex‐mediated formation of ILVs gradually accumulates, leading to the formation of late endosomes [143].

**FIGURE 2:**
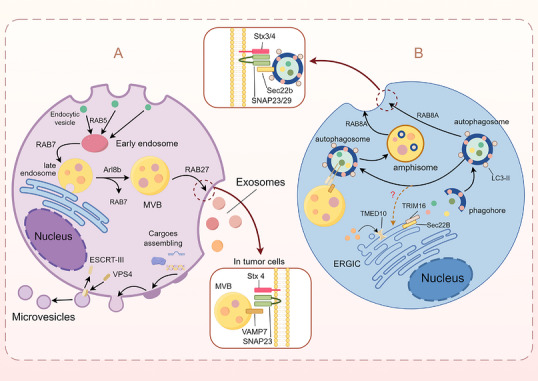
Overview of vesicle‐mediated UcPS pathways. Panel (A) describes UcPS mechanisms in exosomes/MVs. EEs form endosomal vesicles within the ER through the ESCRT mechanism. They are regulated by Rab5/Rab7/Arl8b and mature into multivesicular bodies. These MVBs fuse with the plasma membrane through Rab27 and SNARE complexes, releasing exosomes. MVs bud directly from the plasma membrane, mediated by ESCRT‐III and VPS4. Panel (B) describes the autophagic secretion. TMED10 translocates leaderless cargoes into the ERGIC; TRIM16 binds Sec22b to deliver cargoes to LC3‐II‐positive phagophores, which mature into autophagosomes. Secretion occurs either via SNARE fusion with the plasma membrane, or through intermediate amphisome formation. (Image created with figdraw.com, with permission.)

At the ER‐late endosome membrane contact sites, oxysterol‐binding protein‐related protein 1L regulates the switch from Rab7a to Arl8b, which facilitates the movement of Arl8b‐positive multivesicular bodies (MVBs) toward the plasma membrane and shifts them from a degradative to a secretory phenotype [150]. Rab31 recruits the Guanosine triphosphatase‐activating protein TBC1D2B to inactivate Rab7, thus preventing the fusion of MVBs with lysosomes and promoting the secretion of ILVs as exosomes [151]. Then, MVBs move toward the microtubule‐organizing center through interactions with the actin and microtubule cytoskeleton, before migrating to the plasma membrane and undergoing exocytosis under appropriate cellular stimuli [146, 152]. Rab27a/b plays a critical role at multiple stages of MVB maturation and exosome secretion. Specifically, Rab27b enhances the retention of secretory MVBs at the cell periphery by controlling the transition of MVBs from microtubules to the cortical actin network. Meanwhile, Rab27a inhibits the fusion between these MVBs or with other vesicles, thereby promoting their fusion with the plasma membrane [153, 154]. Interestingly, in epithelial cells, Rab27 only partially regulates apical exosome release, with Rab37 serving as an additional regulator that works alongside Rab27 to control apical exosome release. In contrast, basolateral exosome release is regulated by Rab39 along with its effector protein UACA, forming the Rab39–UACA–BORC complex to mediate this process [155]. In some tumor cells, the fusion of MVBs with the plasma membrane is facilitated by the interaction of two Q‐SNAREs, Syntaxin‐4 and SNAP‐23, with the R‐SNARE VAMP‐7 [156]. Subsequently, the contents of MVBs, namely, ILVs, are released as exosomes into the extracellular environment, where they begin to diffuse until they fuse with target cells (Figure 2A).

The study of exosomes has indeed become extensive, and as mentioned previously, we have broadly covered the formation and secretion mechanisms of exosomes. Now, let us delve into some classic physiological examples to further illustrate these concepts. A study as early as 2007 found that extracellular ATP stimulation could prompt mouse macrophages to release exosomes rich in IL‐1β, caspase‐1, and other inflammasome components [157]. More recently, it has been reported that in intestinal epithelial cells, upon activation of the inflammasome caspase‐8, full‐length GSDMD recruits E3 ubiquitin ligase NEDD4 with the assistance of molecular chaperones Cdc37/Hsp90. NEDD4 catalyzes the polyubiquitination of pro‐IL‐1β, signaling for its loading into secretory vesicles. Subsequently, CD63^+^ small EVs containing these components are released in a GSDMD‐dependent manner; notably, this process does not rely on GSDMD's pore‐forming activity during pyroptosis [158]. Another method of IL‐1β release involves MV‐mediated release, which will be discussed later. In Alzheimer's disease (AD), abnormal phosphorylation of Tau leads to its dissociation from microtubules, forming oligomers [159]. These Tau protein oligomers first pass through the ER and Golgi apparatus before becoming part of secretory vesicles and are ultimately incorporated into exosomes. The reason why Tau proteins opt for this secretion pathway may be related to whether their N‐terminal is cleaved and the involvement of the VAMP8 protein [160]. However, how oligomeric Tau proteins are packaged into exosomes remains unclear. It has also been found that exosomes can transfer pathological tau to unaffected cells, promoting the development of tau pathology within those cells [161]. Regarding α‐synuclein (α‐Syn), which can cause Parkinson's disease (PD), it too can be secreted via exosomes. Specifically, when microglia are stimulated by α‐Syn preformed fibrils, they upregulate the E3 ubiquitin ligase PELI1, mediating the ubiquitination and degradation of lysosomal membrane protein LAMP2, thereby blocking autophagic flux. This results in the abnormal accumulation of autophagosomes that fuse with MVBs, leading to the production and release of exosomes containing α‐Syn (including oligomeric forms) [162].

#### 3.2.2 Microvesicle (Ectosome)

MV (ectosome) is an extracellular vesicle (EV) with a diameter between 100 and 1000 nm. Different from the exosome secretion method, the secretion of MVs occurs through the outward budding of the plasma membrane [163, 164]. The generation of MVs requires multiple molecular rearrangements within the plasma membrane, including changes in lipid composition and protein makeup, as well as alterations in Ca^2+^ levels. The Ca^2+^‐dependent enzyme systems, including aminophospholipid translocases (flippases and floppases), scramblases, and calpain, by restructuring the membrane phospholipid asymmetry, expose phosphatidylserine from the inner layer to the cell surface, thereby triggering the physical bending of the membrane and the remodeling of the underlying actin cytoskeleton [165, 166]. Meanwhile, cholesterol enrichment forms lipid microdomains, inducing local membrane bending and protrusion. Cytoskeletal elements and their regulators are also essential for MV biogenesis. The activity of the small GTPase RHO family and RHO‐associated protein kinases is an important regulator of actin dynamics, and they induce MV biogenesis in different types of tumor cells. Cytoplasmic components are specifically enriched into small membrane domains of the inner leaflet of the plasma membrane through membrane‐anchoring modifications such as palmitoylation, isoprenylation, myristoylation, and the formation of higher‐order complexes and are ultimately incorporated into the budding MVs [167, 168]. Once formed, MVs are pinched off and detached from the plasma membrane, and this process is caused by the interaction between actin and myosin and the subsequent ATP‐dependent contraction [169, 170]. The Rho family small G‐protein Cdc42 can bind to the downstream effector Ras GTPase‐activating‐like protein IQGAP1, driving the reorganization of the actin cytoskeleton to promote MV budding and detachment [171]. At the same time, by inhibiting the endocytosis of EGFR and VEGF90K, sustained EGF signaling is maintained, forming a positive feedback loop that promotes the release of MVs [171]. The detachment of MVs may also be triggered by the interaction between VPS4 and ESCRT‐III. The VPS4 protein binds to the CHMP2A–CHMP3 structure in ESCRT‐III through the microtubule‐interacting and trafficking domain. Driven by ATP, it induces the asymmetric contraction and cleavage of its helical filaments, forming a dome‐shaped structure, causing the membrane neck to constrict to the nanoscale and driving cell or virus budding (Figure 2A) [172].

At the functional level, MVs are involved not only in basic physiological processes but also play important roles in pathological mechanisms. Recently, researchers discovered in a study on sepsis that after lipopolysaccharide stimulates microglial cells, mitochondria generate reactive oxygen species (ROS), which activate the NLRP3 inflammasome, leading to the activation of caspase‐1. The activated caspase‐1 cleaves the inactive pro‐IL‐1β into mIL‐1β and selectively packages it into MVs [173]. These MVs rich in IL‐1β are released extracellularly and can act on neurons and induce synaptic damage [173]. The secretion of Tau protein through MVs is also of great concern. As mentioned before, the secretion of the Tau protein under pathological conditions can occur through exosomes. However, studies have also found that the secretion of tau protein under normal physiological conditions mainly occurs through MVs [174]. The HSP‐70 protein has been found to be transported through MVs. It can activate the AKT signaling pathway and inhibit the phosphorylation of mTOR, thus promoting autophagy in HL‐1 cardiomyocytes and reducing apoptosis [175]. In cancer research, the release mechanism of tumor‐derived MVs (TMVs) is also precisely regulated: small GTPases, including ADP‐ribosylation factor 6 (ARF6) and Rab35, can control the release of TMVs by regulating actin‐myosin contraction [176]. Specifically, the activation of ARF6 can promote the phosphorylation of the myosin light chain by activating extracellular signal‐regulated kinase [177]. Rab35 promotes the release of TMVs by regulating the intracellular distribution of the actin‐bundling protein fascin [178].

#### 3.2.3 Autophagosome

Another common pathway is secretory autophagy, which relies on autophagosomes. As early as 1990 and 1999, Anna Rubartelli's study proposed for the first time that IL‐1β does not proceed through the traditional ER–Golgi pathway, but is secreted by Sec autophagosomes and lysosomes [179, 180]. Autophagosomes are components of the cell vacuole system with a double‐membrane structure and are involved in the process of cellular autophagy. Autophagy was initially defined as a “self‐eating” process that allows cells to digest and recycle unnecessary or harmful intracellular components [181]. Secretory autophagy not only represents a way of handling waste but also a mechanism that permits the release of specific signaling molecules into the extracellular space [182]. First, when lysosomal function is inhibited or impaired, the traditional autophagy pathway becomes difficult to proceed. Evidence shows that the drug bafilomycin A1 can inhibit lysosomal acidification, and in the absence of two SNAREs, SNAP29 or VAMP8, the fusion of autophagosomes with lysosomes can be prevented [183]. In addition, it has been found that inhibiting two PI3K kinases, PIKfyve and VPS34, which are crucial for endosomal transport and autophagy, can also disrupt autophagy‐dependent protein turnover and promote the secretion of various autophagic components in the EV fraction [184, 185]. Rab GTPases play a key role in this process. Among them, Rab8 and Rab27A regulate secretory autophagy, while Rab7 directs the transport of autophagosomes to lysosomes for degradation [2, 186, 187]. According to recent studies, we can divide this pathway into two categories: the ER–Golgi intermediate compartment (ERGIC)–secretory autophagosome pathway and the direct, noncanonical autophagosome secretion pathway.

The process of secretory autophagy involves a series of distinct steps: autophagosome formation and cargo recruitment, autophagosome trafficking, and autophagosome–plasma membrane fusion and secretion [188]. The traditional model posits that proteins enter the interior of autophagosomes through engulfment by the autophagic membrane. However, in the ERGIC–secretory autophagosome pathway, proteins enter the lumen of vesicular intermediates via translocation, eliminating the need for autophagosome closure [189]. Cargo recruitment may be mediated by transmembrane Emp24 domain‐containing protein 10 (TMED10). Initially, with the assistance of heat shock protein 90A (HSP90A), UcPS cargo is unfolded and then binds to TMED10 [134]. TMED10 acts as a transporter, aiding these signal peptide‐lacking cargos to enter the ERGIC. The ERGIC is a tubulovesicular structure located between the ER and Golgi, serving as a compartment marked by unique proteins (such as rat p58 or human ERGIC‐53) [190, 191]. For IL‐1β, initially, it recognizes and binds to HSP90A via two KFERQ‐like motifs, specifically 132–133AA and 198–199AA [189]. Subsequently, it undergoes unfolding, thereby exposing its motif‐1. Following this, the C‐terminal tail of TMED10 binds to this motif with the assistance of its GOLD domain, and oligomerization occurs (i.e., TMED10 oligomerizes) [134]. Small GTPase Rab1 (including Rab1A and Rab1B) promotes TMED10 oligomerization upon binding to GTP, thereby enhancing the transport activity of TMED10 [192]. Subsequently, oligomerized TMED10 translocates with the help of HSP90B1 [134]. Moreover, Rab2A regulates ERGIC partitioning in cooperation with KIF5B, a microtubule‐associated motor protein, forming a UcPS‐specific compartment. Notably, knocking down KIF5B does not affect the entry of signal peptide‐lacking cargos into the ERGIC, indicating its primary role lies in later vesicle transport or partitioning steps [192]. TMED10 is not limited to mediating transport to the ERGIC; instead, galectin‐9 can be transported to ATG9A vesicles via TMED10 for subsequent secretion. This also represents the first discovery that ATG9A, a core autophagic protein, can regulate protein secretion in an autophagy‐independent manner [193]. Recent studies have also demonstrated that the human‐derived TMED family proteins, namely, TMED1‐5, 7, 9, and 10, are all capable of translocating leaderless proteins and differentially regulating the secretion of active UcPS cargoes. The cargo selectivity is mainly achieved through the binding of specific cargoes by the cytoplasmic tails (CT) of TMEDs. Additionally, the ERGIC is an essential site for TMED‐mediated translocation and release. Moreover, the homo‐oligomerization of TMEDs enhances translocation efficiency, and after binding to cargoes, it further stabilizes the oligomers to form a feed‐forward mechanism. Meanwhile, it has also been found that mutations in cargoes such as PCBD1 disrupt the binding to TMEDs and lead to abnormalities in UcPS [194].

The Sec22 homolog B (Sec22B), a longin R‐SNARE, serves as a marker of the ERGIC during the LC3 lipidation process and plays a role in the vesicle and membrane fusion events that occur during the progression of secretory autophagy [195, 196]. Regarding IL‐1β, TRIM16 binds to the secretory autophagy cargo and forms a complex with Sec22b, transferring this cargo to LC3‐II^+^ membrane 25 k carriers [197]. As R‐SNAREs on the cytoplasm‐facing limiting membrane, Sec22b molecules cooperate with Qbc‐SNAREs SNAP‐23 and SNAP‐29 on the plasma membrane as well as plasma membrane Qa‐SNAREs syntaxin 3 and syntaxin 4 to promote fusion [197]. In addition, Rab8A can facilitate the targeted sorting of cytoplasmic proteins to the plasma membrane and their secretion through the secretory autophagy pathway. Rab11 mediates the fusion of MVBs with autophagosomes containing annexin A2 to form amphisomes. Furthermore, Rab8A and Rab27A can also regulate the fusion of amphisomes with the plasma membrane, leading to their release [198].

Amphisomes act as key carriers in secretory autophagy, mediating UcPS by integrating the autophagosome and endosome systems [199]. For example, in intestinal goblet cells, amphisomes labeled with LC3B and endosomal markers (EEA1, Rab7, Rab11) regulate the secretion of mucus granules through ROS signals to maintain the integrity of the intestinal barrier [200]. In IFN‐γ‐stimulated lung epithelial cells, ATG5‐dependent amphisomes coordinate the secretion of annexin A2 through the Rab11–Rab8A–Rab27A GTPase cascade, involving autophagosome–MVB fusion and subsequent plasma membrane docking [201]. The secretion of IL‐1β requires the formation of autophagosome–MVB hybrid structures [189], and ATG5 promotes their fusion with the plasma membrane by inhibiting MVB acidification (by blocking V‐ATPase activity), thus coordinating autophagy–exosome secretion [202, 203]. The cargo sorting mechanism involves ESCRT components (such as TSG101), loading ubiquitinated substrates like HMGB1 into amphisomes, while TRIM family proteins function as cargo receptors [204, 205]. These pathways are activated under stress conditions (oxidative stress, pathogen infection), enabling amphisomes to play a dual role: supporting host defense (such as the secretion of antimicrobial proteins) and also being utilized in pathogenic processes (inflammatory cytokine spread or pathogen release) (Figure 2B) [206, 207].

**FIGURE 3:**
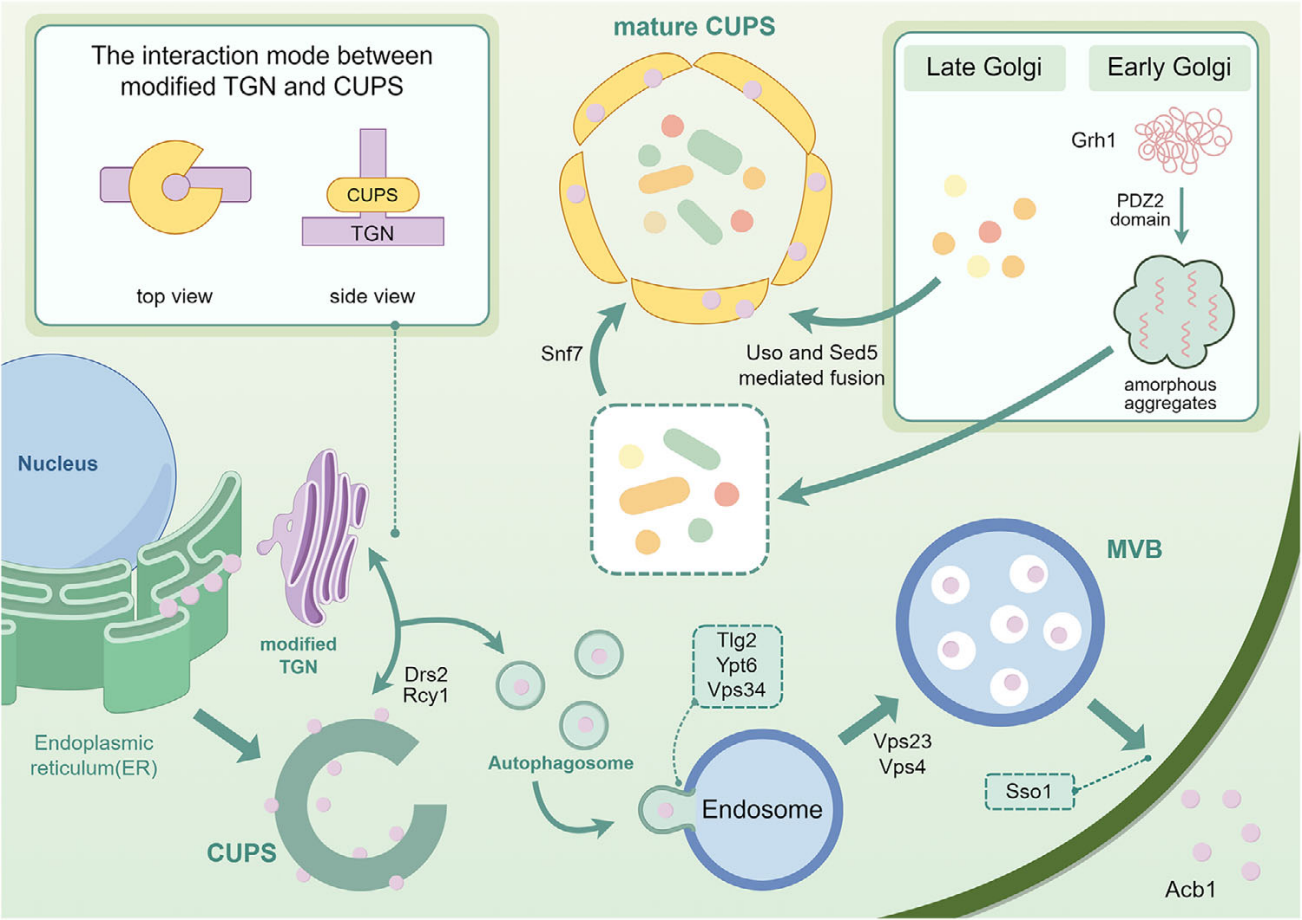
Mechanism of Acb1 unconventional secretion mediated by CUPS in yeast. Unconventional secretion of Acb1 starts with the LLPS of Grh1 in the early Golgi mediated by its SRP domain. Subsequently, early Golgi vesicles and late Golgi vesicles dependent on Sec7 and Pik1 fuse via Uso1 and Sed5 to form CUPS, with the modified TGN providing membrane components for CUPS through its unique interaction mode and Snf7 of ESCRT‐III promoting its maturation. After that, Acb1 is sorted into autophagosomes, fuses with endosomes containing Tlg2 and Ypt6, as well as MVBs, and finally, MVBs fuse with the plasma membrane mediated by Sso1 on the plasma membrane, secreting Acb1 to the extracellular space.

In yeast, the secretion of Acb1, the homolog of acyl‐CoA binding protein that lacks a conventional ER signal sequence, under starvation conditions, is also of great interest. Its secretion requires the involvement of autophagy‐related genes, ESCRT components, and a novel compartment. The basic unit of Compartment for Unconventional Protein Secretion (CUPS) is composed of two types of membrane fusion events: the first type is early Golgi membranes containing Grh1, Bug1, the membrane anchor factor Uso1, and the t‐SNARE Sed5; the second type is late Golgi vesicles dependent on Sec7 and Pik1 (these vesicles are not COP II/COP I vesicles) [208, 209]. This novel compartment is rich in PI3P and phosphatidylinositol 4‐phosphate (PI4P). It neither contains Golgi components (such as coat protein complex I (CopI), Sec7, and Golgi enzymes) nor endosomal components (such as Tlg1 and Pep12); instead, it can recruit autophagy‐related proteins ATG8 and ATG9, as well as Vps23 (a component of ESCRT‐I). Therefore, this compartment is regarded as a novel biosynthetic compartment for secretory autophagosomes [209, 210]. Under starvation conditions, the Golgi reassembly stacking protein (GRASP) Grh1 undergoes liquid–liquid phase separation (LLPS) mediated by its serine/proline‐rich (SPR) domain, forming an amorphous assembly. Subsequently, it translocates to the vicinity of ER exit sites containing Sec13, thereby forming primary CUPS that contain Grh1‐positive vesicles and tubules [210, 211]. After that, ESCRT‐I, ‐II, and ‐III will further participate in the production of CUPS, in which ESCRT‐III subunit Snf7 will contact Grh1 to promote the maturation of CUPS [212]. Notably, although it has been reported that GRASP55, the mammalian homolog of Grh1, can undergo dephosphorylation and relocalization to autophagosomes and MVBs due to mTORC1 inactivation when cells are exposed to stresses such as nutrient deprivation and hypoxia, thereby driving the UcPS of specific cargoes [213]. Whereas GRASP also plays a role in degradative autophagy [214]. This makes it difficult to specifically determine how it distinguishes between secretory autophagy and degradative autophagy. In the late Golgi, Pik1 and Sec7 can promote membrane export by regulating vesicle formation [208]. These vesicles fuse with CUPS via the vesicle tethering protein Uso1 and the Golgi t‐SNARE Sed5 located on CUPS. Acb1, on the other hand, is proposed to enter CUPS by attaching to the saccule that encloses Grh1‐containing membranes, thereby contributing to the formation of mature and stable CUPS [208, 212]. Notably, ESCRT‐0 and the Vps4 AAA‐ATPase are not involved in its formation, which indicates that classical MVBs do not participate in the secretion of Acb1. Interestingly, however, the deletion of Vps4 promotes the earlier localization of Snf7 to CUPS and may be associated with the accelerated secretion of Acb1 [212]. In addition, Drs2, a PI4P effector localized to the trans‐Golgi network (TGN) that acts as a phospholipid flippase and whose ability to support CUPS formation is lost following inactivation by the D560N mutation, together with its interacting protein Rcy1, constitutes a key factor for CUPS maturation. Deletion of either protein results in CUPS vesiculation and impairs its normal sorting function [209]. The sorting of Acb1 depends on its conserved di‐acidic motif DE23/24 (corresponding to DE77/78 in the SOD1 protein) [215]. This motif is proposed to interact with another specific binding partner, which targets cargo proteins to the correct compartment [216]. CUPS also establishes transient contacts with the modified TGN, as evidenced by the colocalization of Drs2 and Grh1 in 26% of cells during early starvation. The modified TGN provides membrane components to CUPS by inserting tubules into it, further supporting the sorting of Acb1 [209]. After sorting, cytoplasmic Acb1 is sequestered into double‐membraned autophagosomes, relying on the coordinated action of the autophagy‐related genes ATG5, ATG7, ATG8, and ATG12. It is then transported via endosomal compartments, mediated by the Rab GTPase Ypt6 and the t‐SNARE Tlg2 (localized to endocytic compartments) [217]. Subsequently, dependent on Vps23 and Vps4 (components of the ESCRT pathway), Acb1‐containing autophagosomes are further fused into MVBs [217]. Finally, MVBs fuse with the plasma membrane under the mediation of the plasma membrane‐localized t‐SNARE Sso1, releasing Acb1‐containing vesicles into the extracellular space and completing the secretion process (Figure 3) [217].

Research on direct, noncanonical autophagosome secretion pathways primarily focuses on the LC3‐dependent EV loading and secretion (LDELS) pathway and the secretory autophagy during lysosome inhibition (SALI) pathway. The LDELS pathway is an inherent pathway in the basic state of cells. It does not require abnormal lysosomal function, and classical autophagy activators (such as rapamycin) can inhibit its activity. It is mainly involved in LC3‐dependent EVs [218]. First, under the action of the LC3‐conjugation machinery composed of ATG7, ATG12, and ATG3, LC3‐B (the core isoform studied) is lipidated from its soluble cytoplasmic form (LC3‐B‐I) to the membrane‐bound active form (LC3‐B‐II). Instead of participating in autophagosome formation during canonical autophagy, this LC3‐B‐II specifically localizes to the limiting membrane of MVBs—a process dependent on ATG7 but independent of ATG14, an initiation molecule of canonical autophagy. Subsequently, RNA‐binding proteins (RBPs, e.g., HNRNPK, SAFB) specifically bind to LC3‐B‐II on the MVB membrane via their intrinsic LC3‐interacting regions (LIR motifs); meanwhile, these RBPs carry small noncoding RNAs (e.g., snoRNAs, miRNAs) to achieve selective cargo capture. Then, neutral sphingomyelinase 2 (nSMase2), recruited by the FAN protein (which binds to LC3‐B‐II via its LIR motif and functions as an adaptor molecule linking LC3 and nSMase2), catalyzes the production of ceramide. Ceramide alters the membrane curvature of MVBs and promotes inward membrane budding, encapsulating the “LC3‐B‐II–RBPs–RNA” complex into ILVs. Finally, MVBs containing ILVs fuse with the cell membrane, releasing ILVs into the extracellular space to form small EVs. These EVs carry intact “LC3‐B‐II–RBPs–small noncoding RNAs” complexes, which can be internalized by recipient cells to mediate intercellular communication [218]. Notably, the types of secreted cargo in this pathway are not limited to RBPs. Recent studies have also shown that the transmembrane protein TFRC can be secreted through this pathway. Different from the secretion process of RBPs, TFRC no longer requires nSMase2 but needs specific ESCRT‐associated components, including PDCD6IP and HGS [219]. The entire process is independent of the degradative function of canonical autophagy and constitutes a specific secretion pathway mediated by the autophagic machinery [218].

When lysosomal function is inhibited or autophagosome maturation is blocked, secretion occurs through another mechanism called “SALI” [183]. Precisely speaking, this pathway is a “branch function” of the classical autophagy pathway when the lysosome is damaged, rather than a completely new pathway independent of classical autophagy. This is because it shares many key autophagy‐related proteins, such as ATG proteins, with the classical autophagy pathway [220]. Classical autophagy induction is directly controlled by TOR kinase [221]. Under abnormal conditions, TOR inhibition causes rapid ATG13 dephosphorylation, allowing ATG13 to interact with ATG1 and ATG17–ATG29–ATG31, promoting ATG1 complex assembly [222, 223]. This complex forms a preautophagosomal structure (PAS) via LLPS [224]. Phagophores emerge in cytoplasm during autophagy, involving ULK1, VPS34, mTOR, ATG5/7/8/9, Beclin‐1, LC3, and so on [225]. Among them, Rab2 assists in the assembly of phagophores [226]. Rab37 promotes ATG5‐12‐16L1, which lipidates LC3 to drive phagophore closure [227, 228]. PAS recruits ATG9 vesicles (with ATG8‐PE/LC3B‐PE) for initial phagophore generation [229]. ATG2–ATG18 and ATG9 (ER‐derived phospholipids) enable expansion [230, 231, 232, 233, 234], and ATG8 lipidation shapes membranes [235, 236]. The upstream initiation stage of SALI, that is, the process before autophagosome closure, is basically the same as the classical autophagy method described above. The main difference between the two lies in the subsequent stages. Classical autophagy leads to degradation, while SALI leads to secretion. In the SALI pathway, the formed complete autophagosomes can form amphisomes with late endosomes. Subsequently, Rab27a can regulate the movement of phagosomes toward the cell membrane and their fusion with it. Eventually, the autophagy cargo receptors and LC3‐II in the phagosomes are secreted extracellularly in the form of binding to the surface of EVs or forming free nanoparticles, along with EVs and particles. This achieves the compensatory clearance of intracellular cargoes destined for degradation, thus maintaining proteostasis [183].

Furthermore, although the misfolding‐associated protein secretion (MAPS) pathway is not directly mediated by autophagosomes, relevant research on it can still help us understand the secretion mechanisms of these misfolded proteins. The MAPS pathway is a protein quality‐control pathway regulated by the deubiquitinase USP19. The USP domain of USP19 has chaperone activity independent of Hsp90, allowing it to preferentially recognize misfolded or unassembled abnormal proteins in the cytoplasm and recruit these abnormal proteins to the surface of the ER via its TMD. Depending on its C506 site, USP19 deubiquitinates these misfolded proteins. Subsequently, the deubiquitinated abnormal proteins are enclosed in ER‐associated late endosomes (marked by the positive marker Rab9), and are finally secreted extracellularly through the fusion of late endosomes with the plasma membrane [237]. The secretion of misfolded proteins is not solely regulated by USP19; for instance, the unconventional secretion of α‐Syn is coregulated by USP19 and UFM1. UFM1 can bind to the CS domain of USP19 to antagonize the activity of its catalytic domain, and together they participate in the regulation of substrate selectivity during the unconventional secretion of α‐Syn. Notably, this process is independent of ubiquitination [238]. Additionally, the secretion of α‐syn may also be mediated by DNAJC5. DNAJC5 anchors to the late endosomal membrane via palmitoylation of its cysteine‐string (CS) domain, and further forms SDS‐resistant, nondisulfide bond‐mediated oligomers. These oligomers can actively translocate cytosolic α‐syn into the endosomal lumen; subsequently, as MVBs fuse with the cell membrane, α‐syn is secreted in the form of soluble monomers [239]. Whether there is a corresponding link between these two secretory pathways is still unknown.

Through the secretory pathway mediated by autophagosomes, various functional proteins such as inflammatory factors, tumor‐related factors, hormones, and so on can achieve UcPS. Autophagy can redirect proteins originally destined for the degradation pathway to the Sec pathway. The mIL‐1β produced by inflammasome activation is recognized and bound by the Sec autophagy receptor TRIM16. TRIM16 localizes to damaged lysosomes by binding to galectin‐8, and at the same time interacts with ATG8 protein and core autophagy proteins to recruit IL‐1β to the autophagosome membrane structure [197]. The chaperone HSP90 assists in the unfolding of IL‐1β and its transport to the intermembrane space of the autophagosome [189]. Subsequently, TRIM16 binds through SNARE and is released through the Sec22b pathway mentioned above [197]. This mechanism also applies to the secretion of other key proteins. The HMGB1 protein mentioned in the previous paragraph can directly bind to HSP90AA1 under stress conditions and is transported from the nucleus to the cytoplasm mediated by XPO1. HMGB1 in the cytoplasm activates the autophagy mechanism and is encapsulated into autophagosomes under the action of factors such as GORASP2, ARF1Q71L, and SAR1A. When the fusion of autophagosomes with lysosomes is blocked by late‐stage autophagy inhibitors, the autophagosomes containing HMGB1 turn to fuse with MVBs. The maturation of MVBs is completed through the ESCRT complex and the ceramide‐dependent pathway. Finally, Rab8A, Rab11A, Rab27A, and so on mediate vesicle transport to secrete HMGB1 out of the cell through the cell membrane [204]. Matrix metalloproteinase 9 (MMP9) is an important factor in the dynamic balance of the ECM, and its secretion mainly depends on the chaperone protein FKBP51. Stress first induces lysosomal damage, promoting an increase in the expression of damage markers such as galectin‐8. FKBP51 indirectly interacts with galectin‐8 by binding to HSP90, recruiting TRIM16 and its loaded MMP9 to the autophagosome. Subsequently, FKBP51 binds to the R‐SNARE protein Sec22b and Q‐SNARE proteins (such as SNAP23, SNAP29, STX3/4) in the Sec autophagy pathway, promoting the assembly of the RQ‐SNARE complex formed by the fusion of autophagosomes with the plasma membrane, and finally releasing MMP9 extracellularly [240]. Similarly, tissue inhibitor of metalloproteinase 1 (TIMP1) is a multifunctional protein that, in addition to acting as an inhibitor of metalloproteinases, also plays a role in promoting cell growth, proliferation, and survival. Research shows that starvation can promote the expression of LC3‐II protein and the release of TIMP1 by activating Rab37, and Sec22b increases the colocalization of Rab37 with autophagosomes, further enhancing the secretion of TIMP1 [241]. In terms of hormone secretion, under high‐glucose stimulation, high glucose can induce the lipidation of LC3 to form LC3‐II. At this time, insulin granules either fuse with LC3^+^ autophagosomes or are encapsulated by them. The small GTPase Rab37 is activated, and through its LC3 interaction region motif, it directly binds to LC3, promoting LC3 lipidation and enhancing autophagosome formation. Activated Rab37 anchors on the autophagosome membrane, mediating the migration of autophagosomes to the plasma membrane. By fusing with the plasma membrane, insulin is released extracellularly. This process does not rely on lysosomal degradation function and is regulated by calcium signals, acting in concert with the traditional insulin secretion pathway [242]. For the virus SARS‐CoV‐2, its encoded ORF3a and ORF7a proteins can promote virus release by interfering with the host autophagy pathway. ORF3a can bind to VPS39, blocking the interaction between Rab7 and VPS39, inhibiting the fusion of autophagosomes with lysosomes, resulting in abnormal autophagosome accumulation [243, 244]. At the same time, ORF3a can rely on ATG7 and autophagosomes as carriers, through the Rab27A‐mediated secretion pathway, and use EVs to release itself [183]. ORF7a activates caspase 3 to cleave SNAP29, disrupting the fusion process of autophagosomes with lysosomes, causing virus‐containing autophagosomes to be transported to the plasma membrane and fuse with it, ultimately releasing progeny viruses extracellularly [245]. Table 2

**TABLE 2:** Summary of the functions of various Rab proteins in UcPS.

Category	Protein	Function	References
ERGIC‐related functions	Rab1	Promote TMED10 oligomerization, enhancing cargo translocation to the ERGIC	[192]
	Rab2	Rab2A interacts with KIF5B to establish a UcPS‐specific ERGIC compartment.	[192]
Endosome maturation and transport	Rab5	Enhance the synthesis of PI3P by activating Vps34, thereby maintaining the lipid identity of EEs	[143]
		Promote the fusion of endocytic vesicles with EEs by recruiting effectors such as EEA1	[143]
	Rab7	The conversion from Rab5 to Rab7 facilitates endosome maturation.	[143]
		The replacement of Rab7 with Arl8b enables kinesin‐mediated peripheral trafficking of MVBs.	[150]
Extracellular secretion regulation	Rab8	Rab8 can interact with the SNARE VTI1/Vti1a/b, participating in unconventional apical protein transport.	[246]
		It works together with Rab11 to participate in the movement of endosomal structures containing apical UcPS cargo toward the apical side.	[246]
		Together with Rab27a, it participates in controlling the fusion of ANXA2‐containing amphisomes with the plasma membrane, promoting the release of ANXA2‐containing exosomes.	[201]
	Rab11	Recruit RABI‐8 to reduce the guanine nucleotide exchange activity of SMGL‐1/GEF on Rab‐8	[246]
		Cooperate with Rab8 to participate in the apical movement of endosomal structures containing UcPS cargo	[246]
	Rab27	Rab27a, along with Rab8a, is involved in controlling the fusion of ANXA2‐containing amphisomes with the plasma membrane, promoting the release of exosomes containing ANXA2.	[201]
		The GTPase conversion from Arl8b to Rab27 promotes the fusion of MVBs with the PM.	[150]
	Rab 37	Additional regulators of apical exosome release in epithelial cells	[155]
	Rab39	The formation of the Rab39–UACA–BORC complex specifically mediates the release of basolateral exosomes in epithelial cells.	[155]
Specific structures formation	Rab31	Drive the formation of ILVs through an ESCRT‐independent pathway	[151]
	Rab35	Recruited to the migrasome formation site by PI(4,5)P_2_, it promotes the formation of migrasomes	[247]
Autophagy‐related functions	Rab8	Participate in the autophagy‐mediated secretion pathway of IL‐1β	[2]
	Rab11	Regulate the fusion of autophagosomes with MVBs to form the amphisome	[248]
	Rab37	Rab37 interacts with ATG5 to promote autophagosome formation.	[228]
		Rab37‐dependent secretory autophagy participates in TIMP1 secretion in lung cancer.	[249]
		Rab37‐dependent secretory autophagy promotes insulin secretion to maintain the homeostasis of insulin and glucose.	[242]

#### 3.2.4 Golgi Bypass

Under conditions of ER stress or mechanical stress, some proteins can also be secreted, bypassing the Golgi apparatus. Unlike the types mentioned above, these proteins still possess signal peptides. These signal peptides are recognized by the SRP, which recruits them to the ER during protein synthesis by ribosomes. Nevertheless, different from the classical secretory pathway, this route does not involve processing through the Golgi apparatus but directly transports proteins to the extracellular space via vesicles.

Usually, many secretory proteins possess N‐ and/or O‐linked oligosaccharide chains that undergo complex modification reactions [250]. These proteins that bypass the Golgi apparatus exhibit an immature ER core–glycosylation pattern. Therefore, the presence of proteins with ER core–glycosylation on the cell surface can serve as a marker for transmembrane proteins that utilize the Golgi‐bypass pathway. By bypassing the Golgi apparatus, proteins can protect themselves and be secreted more rapidly, so as to better respond to external stimuli and rapidly upregulate the expression of proteins on the cell surface [251]. Different proteins may utilize different Golgi‐bypass pathways. The main pathway is mediated by GRASP proteins, but there are also other distinct routes.

GRASP is one of the important sorting mechanisms in the Golgi bypass. GRASP55, a common mammalian GRASP protein, is linked to the cytoplasmic side of the Golgi membrane through myristoylation. This protein was initially recognized as an essential component for the formation of stacked Golgi cisternae in vitro [252, 253]. Previous studies have shown that GRASPs are not essential for most conventional anterograde protein transport [254]. However, they are thought to be involved in many UcPS pathways that bypass the Golgi apparatus. The structure of GRASPs can be divided into two main regions. One region consists of two tandem PSD95/Dlg1/ZO‐1 (PDZ) domains, and the other is the SPR domain [255]. The homodimerization or oligomerization of GRASPs is crucial for the tethering of Golgi membranes and is promoted by the interaction between the two PDZ domains of GRASPs. Under stress conditions, the phosphorylation of Ser441 in the C‐terminal region of GRASP55 promotes the dissociation of the GRASP55 homopolymeric complex into monomers and leads to the relocalization of GRASP55 from the Golgi apparatus to the ER [256]. Then, the GRASP protein relocalizes to the ER and interacts with the abnormally accumulated proteins on the ER, thus initiating the transport through the pathway that bypasses the Golgi apparatus.

For the CFTR, there are usually two transport pathways. The first is the traditional pathway through the Golgi apparatus. During this process, CFTR is synthesized and folded in the ER and then exported from the ER via COPII‐coated vesicles. CFTR undergoes glycosylation modification in the Golgi apparatus to form a mature glycoprotein [257, 258] and is subsequently transported to the TGN via COPI‐coated vesicles. Starting from the TGN, CFTR can be directly transported to the plasma membrane through transport vesicles or transcytosed through the basolateral plasma membrane. It can also first reach the apical recycling endosome (ARE) and then be transported from the ARE to the plasma membrane [259]. The other pathway involves transport from the ER to the plasma membrane via GRASP. When misfolded proteins accumulate in the ER, oligomerized IRE1α activates the ASK1 signaling pathway, thus regulating vesicle secretion [260]. Meanwhile, as mentioned before, monomerized GRASP55 can specifically bind to the C‐terminal peptide of CFTR through its PDZ1 domain, mediating the retention of ΔF508–CFTR in the ER [3, 256]. Then, the tubular ER structure ER–TB, shaped by the ER–phagy receptors ATL3 and RTN3L, enriches the ΔF508–CFTR protein retained in the ER within its own structure by forming a complex tubular–vesicular network. Subsequently, it is transported along microtubules toward the cell surface with the help of microtubule dynamics [261]. During this transport process, with the assistance of two adaptor proteins, FYCO1 and SKIP, two kinesins, KIF1A and KIF5A, can promote the movement of vesicles along microtubules [262]. In hepatocellular carcinoma cells, cytoplasmic proteins first interact with extended‐synaptotagmin 1 (E‐Syt1) on the ER and are incorporated into Sec22b^+^ vesicles. These vesicles then fuse with the plasma membrane through Q‐SNAREs (such as SNAP23, SNX3, and SNX4), thus facilitating protein secretion [263]. The anchoring and stability of proteins on the plasma membrane depend on interactions with various proteins, especially the Na^+^/H^+^ exchange regulatory factor homolog 1, which plays a key role by generating proper anchoring interactions with the cytoskeleton through a multiprotein complex [259].

Other reviews have mentioned that Hsp70 and its cochaperone DNAJC14 represent another sorting mechanism [79], which has so far only been explored in the secretory pathway of the pendrin protein. Specifically, the HSP70 family member Hsc70 acts in concert with the cochaperone DNAJC14. DNAJC14 accelerates the ATPase activity of Hsc70 through the HPD motif in its J domain, enabling the formation of a complex between the two. This complex then binds to misfolded pendrin (such as H723R pendrin), preventing it from being degraded by the ER‐associated degradation pathway. Subsequently, this complex guides pendrin to bypass the Golgi apparatus via an unconventional Sec pathway that depends on Rab18‐positive vesicles, thus facilitating the secretion of misfolded pendrin [264].

COPII vesicles, which participate in the conventional secretory pathway, have also been found to be involved in this unconventional pathway. Peripherin/rds, a key integral membrane protein in the outer segment disk membranes of retinal photoreceptors, achieves its transport through a Golgi bypass secretion route. After being synthesized and initially N‐glycosylated in the rough ER, it buds off from the ER in a COPII‐dependent manner to form transport vesicles that bypass the Golgi apparatus without relying on the Golgi‐associated protein GRASP55, directly targeting the connecting cilium (a structure similar to primary cilia) [265]. Experimental evidence shows that its glycosylation state is sensitive to endoglycosidase H [265], indicating that it has not undergone processing by mannosidase II in the Golgi. Moreover, Golgi disruptors such as brefeldin A and monensin do not affect its transport [265], further confirming that the pathway is independent of the Golgi apparatus. During transport, diacylglycerol kinase may indirectly regulate its exit from the ER by promoting COPII vesicle formation [266]. This unconventional secretion pathway ensures efficient targeting of peripherin/rds to the photoreceptor outer segments, with abnormalities in this mechanism potentially leading to retinal degeneration, providing an important model for studying the pathology of ciliary protein transport.

Transport is mediated through mixed‐identity organelles. These organelles incorporate components of the TGN, late endosomes, or lysosomes, such as TGN38/46, GM130, giantin, and other Golgi‐associated proteins, along with lysosomal marker protein LAMP1 and vesicle fusion protein VAMP7 [267]. In axons, after transmembrane proteins are synthesized and N‐glycosylated in the smooth ER [268], they bypass the conventional Golgi body modifications and instead enter mixed‐identity organelles. These organelles dynamically assemble to form vesicles that directly transport proteins to the axonal cell membrane via a membrane fusion mechanism mediated by vesicle‐associated membrane protein VAMP7 [269]. For instance, the TRPM8 ion channel colocalizes with LAMP1 and Rab7 in heterologous systems, completing secretion through lysosome‐like vesicles [269]. Similarly, after the L1 cell adhesion molecule undergoes endocytosis into Rab11‐positive endosomes, it is also resecreted to the cell membrane via a VAMP7‐dependent pathway [270, 271], circumventing the traditional processing steps of the Golgi apparatus. This process achieves local transport and functional integration of proteins.

## 4 Prokaryotic‐Specific Secretion Mechanisms

To date, as many as 11 types of specialized secretion systems have been identified in prokaryotes [6]. Among these systems, four are considered to share similarities with the UcPS in eukaryotes, in that the proteins secreted by these systems do not require signal peptide mediation. These four secretion systems are T1SS, Type III secretion system (T3SS), Type VI secretion system (T6SS), and Type VII secretion system (T7SS), respectively. In the following sections, each of these four secretion systems and their associated pathogenicity will be elaborated on in detail.

### 4.1 Secretion Systems of Prokaryotes

The canonical T1SS is composed of three core components: an IM ABC transporter, a periplasmic adaptor protein (PAP), and an OM TolC family OM factor (OMF) [272]. As described in detail earlier, the ABC transporter functions here to energize the secretion process and specifically recognize secretion substrates [272]. In addition, the PAP serves to bridge the components of the inner and outer membranes and transduce the secretion signal [273, 274, 275], while the OMF forms a gated channel across the OM. This channel remains in a closed state at rest to prevent nonspecific leakage, and opens upon activation to form a continuous transport pathway [276, 277, 278]. The archetypal T1SS, represented by the Escherichia coli hemolysin A (HlyA) system, adopts a one‐step secretion mechanism. The ABC transporter and PAP are constitutively preassembled in the IM [279, 280]. Upon recognition of the noncleaved C‐terminal secretion signal of the substrate [281], the complex recruits the OMF to assemble a continuous channel spanning the entire cell envelope [280]. Energized by ATP hydrolysis, the system secretes the unfolded substrate directly from the cytosol into the extracellular milieu [282]. The substrate then folds and acquires its biological activity in the high‐calcium extracellular environment, and the secretion system dissociates and resets to its resting state upon completion of secretion [282, 283]. Recent research has subdivided T1SSs into five subgroups. Some subgroups can anchor substrates on the bacterial cell surface and even harbor a two‐step secretion mechanism, which breaks through the traditional understanding of this secretion system [284].

The T3SS is a needle‐like complex whose core function is to deliver bacterial effector proteins into the cytoplasm of eukaryotic cells. In the T3SS, a sorting platform (SP) is formed by a complex of soluble cytoplasmic proteins, including SctQ, SctK, SctL, SctN, and a smaller amount of SctO, which may help establish a hierarchical secretion order [285, 286]. Among these, SctQ, SctK, and SctL are essential structural components of the platform [287], whereas SctO and SctN are required for secretion but play only auxiliary structural roles [287, 288]. Initially, the regulatory protein SctP binds to the SP, enabling it to preferentially recruit early substrates such as SctI (which forms the inner rod of the needle complex [NC]) and SctF (the needle filament protein) [286, 289]. These substrates are unfolded by the ATPase SctN [290], secreted through the export apparatus, and assembled into the NC. Subsequently, SctP dissociates, switching the platform to a “middle substrate mode” [291]. Subsequently, SctP dissociates, switching the platform to a “middle substrate mode” [292], which senses the presence of a suitable target cell and triggers the secretion process in the bacterial cytoplasm. Next, middle substrates (the translocators SctE and SctB), assisted by their chaperone SctW, bind to the platform [286]. After unfolding, they are secreted through the NC and form pores in the host membrane, creating a transmembrane channel known as the translocon [293]. Finally, the platform switches to a “late substrate mode.” Late substrates (effector proteins), assisted by dedicated chaperones (which bind last due to their lowest affinity), interact with the platform. These effectors are unfolded in an SctN‐dependent manner  and delivered into the host cell cytoplasm via the translocon [290]. Finally, the platform switches to a “late substrate mode.” Late substrates (effector proteins), assisted by dedicated chaperones (which bind last due to their lowest affinity), interact with the platform. These effectors are unfolded in an SctN‐dependent manner and delivered into the host cell cytoplasm via the translocon [294, 295].

The T6SS shares certain similarities with the T3SS, as both facilitate direct secretion by penetrating target cell membranes. More precisely, the T6SS functions like a bacteriophage that pierces prey cells to inject its DNA, thereby penetrating the bacterial cell envelope [296, 297]. The T6SS is primarily composed of three structural parts. The membrane complex (TssJ, TssL, TssM) spans the inner and outer membranes and has no homolog in phage systems [298]. In contrast, the remainder of the system closely resembles the contractile tail of bacteriophages. A heteromeric ring‐like complex termed the baseplate (composed of TssA, TssE, TssF, TssG, TssK) is assembled on the membrane complex [299, 300, 301, 302]. This baseplate serves as the assembly site for the contractile sheath (formed by TssB and TssC) [303, 304], which polymerizes in the cytoplasm as a 12‐stranded helical tube that encapsulates a stack of hexameric Hcp rings [305]. The baseplate surrounds a puncture device, consisting of a trimeric, cone‐shaped VgrG protein [297]. The flat side of the VgrG trimer interacts with the first Hcp ring  [305], while the tip of the cone is sharpened by a PAAR‐repeat protein [306]. The contraction of the T6SS is triggered once the fully assembled sheath extends across the cytoplasm and contacts the membrane opposite the baseplate [299, 302, 307], This process involves a conformational change in the baseplate structure [308], and results in the outward propulsion of the VgrG spike and the Hcp tube through the membrane complex. These components are ejected across the OM through a multimeric pore formed by TssJ [309]. Studies in Pseudomonas aeruginosa have demonstrated that the primary role of the T6SS is to function as a nanomachine that delivers toxin effectors into bacterial competitors, leading to their elimination [310].

The T7SS, also referred to as the ESAT‐6 protein family secretion system, in mycobacteria is capable of translocating a variety of virulence factors across the cell membrane. Substrates of the T7SS—including Esx, PE/PPE, and Esp proteins—must first be recognized by the system via their conserved WXG and YXXXD/E motifs (where X represents any amino acid) [311, 312]. Prior to transport, these substrates are prepared in the cytoplasm with the assistance of molecular chaperones such as EspH [313], EspG [314, 315], and EccA [316]. Translocation across the IM is then driven by conformational changes and energy input from the membrane complex, composed of EccB, EccC, EccD, EccE, and MycP [317]. Three possible models have been proposed for this IM transport: the central pore model, the agitator model, and the protomer pore model [318]. The central pore model is the most widely accepted. It proposes that an Esx substrate initially binds to the third NBD of EccC, promoting its hexamerization. Subsequently, the LINKER‐2 region interacts with a PE/PPE protein or its chaperone, relieving inhibition of NBD1 and thereby activating the membrane complex. This triggers a conformational change in EccC from an extended state to an ATPase‐active “bucket” state, which opens the pore of the membrane complex and enables substrate translocation across the IM [318]. The core membrane complex of the T7SS is not sufficient to span the bacterial OM. Current research hypothesizes that the translocation across the OM may be mediated by the substrates themselves [319, 320], although the underlying mechanism remains incompletely understood. Table 3

**TABLE 3:** Comparative overview of the Type III, Type VI, and Type VII bacterial secretion systems.

	T3SS	T6SS	T7SS/ESX
Structure and composition	Syringe‐like apparatus comprising a membrane‐embedded NC and a cytoplasmic SP [289, 321]	Phage tail‐like contractile structure consisting of a membrane complex, sheath, baseplate, Hcp, VgrG, PAAR proteins, and so forth [299, 300, 301, 302]	Transmembrane complex (EccB/C/D/E, MycP) with cytoplasmic components (e.g., EccA, EspG) [317]
Secretion mechanism	Substrate recognition and ordering by the SP; ATPase (SctN)‐driven, ordered secretion through a central conduit [285, 286, 290]	Sheath contraction propels the spike complex to penetrate the target membrane, resulting in effector delivery [309].	Substrate translocation via proposed models (central pore, agitator, or protomer pore), dependent on the ATPase EccC [318]
Primary function	Injection of effector proteins into eukaryotic host cells to modulate host cell functions, promoting bacterial infection and survival	Interbacterial competition, metal acquisition, virulence delivery, and biofilm formation	Virulence factor secretion, immune evasion, nutrient acquisition, and contribution to antibiotic resistance
Substrate types	Effectors, translocators, needle filament proteins [286, 289, 290]	Toxic effectors (e.g., hydrolases, nucleases, phospholipases), cognate immunity proteins [299, 322, 323, 324]	Esx proteins, PE/PPE proteins, Esp proteins [311, 312]
Target cells	Eukaryotic cells	Prokaryotic cells, eukaryotic cells, fungi	Primarily immune cells
Regulatory mechanisms	Environmental signals, transcriptional control, secretion switch proteins (e.g., SctP); functional networking among effectors [286, 289, 325, 326]	Quorum‐sensing, two‐component regulatory system, pH, temperature changes, c‐di‐GMP [327, 328, 329]	Two‐component systems, transcription factors (e.g., WhiB6) [320, 330, 331]
Evolutionary origin	Homologous to the bacterial flagellar system [332]	Homologous to the bacteriophage tail structure [333]	Likely evolved from the ancestral ESX‐4 system via gene duplication and horizontal gene transfer [317]
Chaperone dependence	Yes, most effector proteins require specific chaperones [334]	Yes, some effectors rely on adaptor or coadaptor proteins [335]	Yes, utilizes chaperones such as EspG, EspH, and EccA [313, 314, 315, 316]
Distribution	Gram‐negative bacteria	Gram‐negative bacteria	Mycobacteria and other Actinobacteria; also identified in some Gram‐positive bacteria
Example pathogens	Salmonella spp., Shigella spp. [287, 336]	Vibrio cholerae, Pseudomonas aeruginosa, Salmonella enterica, Acinetobacter baumannii, Burkholderia spp. [337, 338]	Mycobacterium tuberculosis, Mycobacterium marinum, Staphylococcus aureus [339, 340]

*Abbreviations*: ESX, ESAT6 protein family secretion systems; T3SS, Type III secretion system; T6SS, Type VI secretion system; T7SS, Type VII secretion system.

### 4.2 Pathogenicity

Prokaryotes utilize the T1SS to mediate the direct transmembrane release of unfolded virulence factors, which serves as the core molecular mechanism for pathogens to disrupt host physical barriers and remodel the infection microenvironment. Diverse pathogenic bacteria have evolved distinct pathogenesis strategies via T1SS: Escherichia coli and Moraxella bovis secrete RTX family toxins (e.g., HlyA, MbxA) to directly lyse host cell membranes, leading to their rapid death [341, 342]. Salmonella Enteritidis utilizes its effector SiiE to mediate initial adhesion to and invasion of host epithelial cells [343], and synergizes with SiiD to inhibit the activation of the NLRP3 inflammasome, thereby blocking host immune clearance [344]. Furthermore, T1SS is closely associated with the virulence evolution of pathogens, demonstrating significant gene enrichment in hypervirulent Klebsiella pneumoniae [345]. Whereas in chronic pulmonary infections caused by Pseudomonas aeruginosa, the effector protein TesG specifically impairs alveolar macrophage function, with its expression level being highly correlated with poor patient prognosis [346]. For obligate intracellular parasites such as Rickettsia, their T1SS substrates possess the ability to modulate host transcription or cytoskeletal dynamics, thereby ensuring their intracellular survival while evading innate immune detection [347]. In summary, T1SS is not merely a transport channel for virulence factors but a molecular hub for pathogens to achieve evolution from physical defense disruption to precise immune manipulation.

As a sophisticated multicomponent protein translocation platform, the T3SS directly delivers effector proteins into the host cell to achieve precise regulation of host physiological processes. Its pathogenic logic exhibits remarkable tissue adaptability: in intestinal infections, pathogenic Escherichia coli exploits the effector kinase NleH/CesT axis to precisely regulate colonization dynamics [348]. The Salmonella SPI‐1 system specifically intervenes with mitochondrial respiratory chain complex I to induce host metabolic rewiring while maintaining membrane potential, creating a stable intracellular colonization environment for the bacteria—a process efficiently regulated by SpaO isomers [349, 350]. The Shigella effector IpaB initiates the invasion pathway by mediating translocation across the intestinal epithelial barrier and has become a core target for vaccine development [351]. In systemic infections, Pseudomonas aeruginosa utilizes sRNA102 to target the core T3SS component PcrG for immune evasion [352], and its activity is regulated by the RNA methyltransferase PA3840, which is translocated cross‐system via T6SS [353]. The Xenorhabdus effector XopA targets host tubulin to induce apoptosis, thereby systemically suppressing the host's inflammatory response [354].

Operating as a retractable nanomachinery, the T6SS strikes host cells or competing microorganisms in a contact‐dependent manner, demonstrating profound evolutionary adaptability in breaching physiological barriers and engaging in microbial competition. In central nervous system (CNS) infections, neonatal meningitis Escherichia coli leverages T6SS components (Hcp, ImpK, IcmF) to drive host endothelial cytoskeletal rearrangement and apoptosis, subsequently breaching the blood–brain barrier [355]; whereas in urinary tract and systemic infections, T6SS expression is significantly and positively correlated with the risk of thrombosis induced by Klebsiella pneumoniae [356]. Within complex microecological environments, Salmonella utilizes diversified hcp genes to drive gut microbiota dysbiosis for colonization advantages [357], while Vibrio parahaemolyticus induces macrophage autophagy to enhance its infectivity [358]. Such systems are also highly prevalent in diarrhea caused by Campylobacter concisus isolates [359]. Furthermore, Acinetobacter and Stenotrophomonas maltophilia maintain a competitive advantage in polymicrobial communities by relying on their vast effector repertoires and can promote pathological progression by amplifying host immune response [360, 361].

T7SS is a critical hub for pathogens to evade host immune surveillance, combat environmental stress, and achieve tissue‐specific colonization. In terms of immune evasion, Staphylococcus aureus secretes the EsxB protein via T7SS to precisely antagonize the host STING pathway, thereby suppressing the immune response of macrophages during early infection [362]. Conversely, the T7SS substrates of Mycobacterium tuberculosis serve as the core antigenic targets recognized by host CD4+ T cells during latent infection [363]. Moreover, T7SS is equally crucial for stress resistance and survival, such as protecting Staphylococcus aureus against antimicrobial fatty acids secreted by the host [364], or driving Corynebacterium glutamicum to actively acquire iron resources via the ExsI–ExiR mechanism to counter “nutritional immunity” [365]. In tissue‐specific infections, the genetic heterogeneity of T7SS directly determines the colonization advantage of Group B Streptococcus in the reproductive tract and its virulence level in neonatal early‐onset disease [366, 367]. Meanwhile, the Streptococcus gallolyticus subsp. Gallolyticus effector TelE utilizes a conserved glycine zipper motif to exert cytotoxicity, driving its associated opportunistic pathogenesis [368].

## 5 Emerging Pathways—Migrasome

Migrasomes are a newly discovered type of cellular structure in recent years. Their core function is to assist cells in material transport and intercellular communication during cell migration. Although it is not a subset of UcPS, we believe that understanding it can help us enhance our comprehension of aspects such as vesicle transport in UcPS. Therefore, we will provide a brief explanation of it here. Migrasomes are membranous vesicle structures formed at the ends and intersections of retraction fibers (RFs) during cell migration, with a diameter of 0.5–3 µm. It has been recognized as a distinct, targeted form of protein release, facilitating the secretion of proteins like colony‐stimulating factor‐1 and monocyte chemoattractant protein‐1, as well as damaged mitochondria exocytosis [369, 370, 371]. The development of migrasomes starts with sphingomyelin synthase 2 (SMS2) clustering within migrating cells. SMS2 foci at the cell's leading edge remain on RFs, initiating migrasome formation. Conversion of ceramide to sphingomyelin by SMS2 triggers growth [370, 372]. PIP5K1A is recruited to these sites to catalyze the production of PI(4,5)P_2_, which subsequently recruits Rab35. The Rab35 protein then assembles integrin α5 to bind with the ECM [247, 372, 373]. Migrasomes tightly adhering to the ECM efficiently absorb cytoplasmic contents, initiating expansion via membrane tension on RFs. Migrasomes tightly adhering to the ECM efficiently absorb cytoplasmic contents, initiating expansion via membrane tension on RFs. Syt‐1 induces unstable membrane swelling in a calcium‐dependent manner, which is stabilized by tetraspanin 4 to form mature migrasomes [372, 374, 375]. Similar to MVs, their lumens contain vesicles enriched with CAV1 and Rab10. LRRK2 phosphorylates Rab10 to coordinate Myosin‐5a and RILPL2, transporting these vesicles along RFs [376]. After that, the fusion with the migrasome membrane occurs via v‐SNARE VAMP2 interacting with Q‐SNAREs syntaxin4 and SNAP23 [369].

As an important cellular structure mediating targeted molecular delivery, the functions of migrasomes have extended to cell communication, biological development, and various pathological processes. Research indicates that macrophage colony‐stimulating factor and monocyte chemoattractant protein‐1 (also known as chemokine (C–C motif) ligand 2) form secretory vesicles labeled with VAMP2, which are actively transported to the migrasomes at the rear of the cell via the actin‐dependent motor protein Myosin‐5a [369]. This process relies on the membrane fusion mechanism mediated by the SNARE complex (such as VAMP2 with syntaxin4 and SNAP23) and is released into the migrasomes and then released along with the migrasomes promoted by calcium signals [369]. Further studies have found that p21‐activating kinase 4 and laminin alpha 4, ECM‐related proteins, can also be released through migrasomes. Their secretion can enhance the migration and invasion abilities of glioblastoma (GBM) cells. Knockdown of the TSPAN4 gene can reduce migrasome formation, thereby inhibiting the migration and invasion of GBM cells as well as tumor growth in vivo [377]. In the coagulation system, neutrophil‐derived migrasomes are rich in coagulation factors. Experiments show that incubation with platelets can activate platelets, revealing the key role of migrasomes in coagulation regulation [378]. At the level of biological development, migrasomes also play a central role. Migrasomes produced by monocytes in the chorioallantoic membrane of chicken embryos are rich in factors such as CXCL12, VEGF‐A, and TGF‐β3, promoting endothelial cell tube formation and monocyte recruitment [379]. In zebrafish, the CXCL12 chemokine carried by migrasomes guides the migration of dorsal progenitor cells along the dorsal axis. Loss of its function can lead to organ ectopia or developmental defects [379].

## 6 Biological Functions and Physiological Roles

### 6.1 Intercellular Communication and Signal Transduction

FGF2 is a potent stimulator of growth and differentiation, playing a crucial role in physiological processes such as angiogenesis. Compared with the conventional ER–Golgi pathway, FGF2 secreted via the direct transmembrane transport mechanism can be secreted more rapidly [81]. This may enable rapid and directional signal transduction for physiological processes like angiogenesis and tissue repair, ensuring that cells can respond promptly and adaptively to changes in the microenvironment.

Under physiological conditions, Type III UcPS enables precise intercellular communication and signal transduction through vesicle—mediated extracellular transport mechanisms, mainly including exosomes, MVs, and secretory autophagy. As a key carrier, exosomes can directionally transport α‐Syn between neurons, participating in neural signal transmission [162]. MVs carry the HSP‐70 protein to activate the AKT signaling pathway, inhibit mTOR phosphorylation, promote cardiomyocyte autophagy, and reduce apoptosis [175]. In intestinal goblet cells, amphisomes (carrying LC3B and endosomal markers such as Rab7/Rab11) precisely regulate the secretion of mucin through ROS signals [200]. Additionally, in IFN‐γ‐stimulated lung epithelial cells, ATG5‐dependent secretory autophagy targets and secretes annexin A2 via the Rab8A/Rab11/Rab27A GTPase cascade, affecting the homeostasis of the cellular microenvironment [201].

In addition to the aforementioned mechanisms, the migrasome acts as a unique “spatially localized signal center” in cell communication and signal transduction. Its functions are mainly reflected in two aspects: First, the migrasome specifically enriches key signaling molecules (e.g., CXCL12) and bioactive substances, and undergoes site‐specific deposition along the cell migration trajectory, thereby establishing precise and highly localized spatiotemporal signal gradients in the tissue microenvironment. This patterned distribution overcomes the limitations of conventional diffusible signals, as exemplified by its role in orchestrating the formation of Kupffer's vesicles in zebrafish embryos and directing angiogenesis in chick embryos [7, 380]. Second, the migrasome not only transduces signals through ligand‐receptor binding but also acts as a carrier to directly mediate the lateral transfer of functional cargos (e.g., PTEN mRNA and protein) to recipient cells, thus altering their cellular fates [381], and enables direct contact‐mediated intercellular communication via membrane fusion.

Golgi bypass modulates intercellular communication and intracellular signal transduction through the precise targeted sorting, efficient membrane localization, and retention of core glycosylation modifications of signal‐associated membrane proteins, thereby ensuring the precision, timeliness, and functional diversity of signal transmission under physiological homeostasis. In highly polarized neurons, this pathway serves as a core mechanism for maintaining the homeostasis of synaptic communication: the Kv2.1 potassium channel at the axon initial segment relies on this pathway for membrane localization, which finely regulates neuronal membrane excitability and guarantees the normal initiation of action potentials as well as interneuronal electrical signal communication [382]. Meanwhile, the AMPA‐type glutamate receptor GluA1 and other related proteins on the dendritic postsynaptic membrane achieve dynamic replenishment on the membrane surface via this pathway, which ensures the efficient reception and transmission of synaptic chemical signals and maintains the normal communication of neural networks [383]. In immune cells, a subset of CD45 phosphatase on the surface of T cells reaches the cell membrane through this pathway, and the resulting glycosylation heterogeneity can precisely regulate T cell activation‐related signaling pathways, thus sustaining the communication homeostasis among immune cells [384, 385].

### 6.2 ECM Remodeling

There is a close association between migrasomes and the ECM, and the mechanism underlying their crosstalk remains a critical scientific question that urgently requires in‐depth investigation in this field. Integrins serve as key transmembrane receptors mediating cellular adhesion to the ECM, and their precise pairing with ECM ligands constitutes the core molecular basis for the regulation of migrasome biogenesis. Upon specific binding to their cognate ECM ligands, integrins switch to an activated state and become enriched at the contact sites between RFs at the cell rear and the matrix, stably anchoring these sites to the ECM. This anchorage not only determines the initial site of migrasome formation but also provides a stable tethering force for RFs during forward cell migration, ensuring their proper extension and thereby supporting the subsequent growth and maturation of migrasomes [373]. More notably, migrasomes are not merely passive effectors regulated by the ECM; they can also exert a reverse regulatory effect to remodel the ECM in the microenvironment. In GBM models, existing studies have revealed that PAK4 and LAMA4 can be specifically enriched in migrasomes. Following the extracellular release of migrasomes and the lysis of their contents, these two molecules are secreted into the tumor microenvironment, where they significantly enhance the migratory and invasive capacities of surrounding GBM cells by modulating ECM remodeling and cell adhesion processes [377].

During specific stages of epithelial developmental remodeling in Drosophila, integrin αPS1 can be transported to specific plasma membrane domains through a dGRASP‐mediated pathway that bypasses the Golgi apparatus, and this process plays a critical role in the establishment of de novo basal adhesions and the maintenance of epithelial structural integrity [254]. In colon carcinoma cells, membrane Type‐1 MMP (MT1‐MMP) can also achieve rapid cell surface trafficking via a Golgi‐bypassing unconventional pathway, thereby mediating ECM degradation and facilitating tumor invasion [386]. Another MMP, MMP9, can be secreted via secretory autophagy in response to glucocorticoid stimulation. The enhanced secretion of MMP9 increases the cleavage of pro‐BDNF (pro‐brain‐derived neurotrophic factor) into its mature form, mBDNF (mature brain‐derived neurotrophic factor), thus further promoting synaptic plasticity [240]. Interestingly, however, some studies have reported that in lung cancer cells, active RAB37 and autophagy act together to enhance the efflux of TIMP1 through the secretory autophagy pathway. TIMP1 is known to inhibit MMP9, thereby suppressing the migration and metastasis of lung cancer cells [241]. Both of these can be secreted through the process of secretory autophagy. Whether there are specific associations and reciprocal regulations between them under normal physiological conditions remains unknown.

## 7 Disease Connections and Therapeutic Opportunities

The involvement of UcPS in disease pathogenesis is increasingly recognized, yet moving beyond mere association to establish causative mechanisms requires critical examination. While UcPS pathways provide a compelling explanation for the release of key pathological factors, the field must carefully dissect whether UcPS is a primary driver of pathology, a passive consequence of cellular stress, or an attempted compensatory response.

### 7.1 Cancer and Metastasis

In the tumor microenvironment, EVs play a crucial role by delivering various miRNAs, maintaining cancer stem cell characteristics, and remodeling the tumor stroma to support tumor growth and invasion, thereby promoting tumor immune escape and metastasis [387]. In GBM, HMGB1 secreted through autophagy pathways enhances tumor‐associated macrophages (TAM) M1‐like polarization via the RAGE–NF‐κB–NLRP3 inflammasome pathway, thus increasing the chemosensitivity of GB cells to temozolomide [388]. The Rab37–Sec22b pathway inhibits matrix degradation by secreting TIMP1, thereby limiting lung cancer metastasis [241]. E‐Syt1 mediates the secretion of a key cytoplasmic protein at ER–PM contact sites in hepatocellular carcinoma cells. This process is spatially confined to the Sec22b vesicle transport route, facilitating the secretion of protein kinase C δ (PKCδ) through an unconventional secretion pathway involving Q‐SNAREs (SNAP23, SNX3, and SNX4). Importantly, PKCδ antibodies effectively inhibit its secretion and oncogenicity, providing a foundation for targeted therapy in liver cancer [263]. As a classic tumor suppressor gene, PTEN has been found in recent years to undergo unconventional secretion via the TMED10‐channeled UcPS (THU) pathway, which is mediated by the specific binding of its W274 amino acid residue to TMED10. Extracellularly secreted PTEN functions as a cytokine‐like molecule. By binding to the PLXDC2 receptor on the surface of macrophages, it activates the JAK2–STAT1 signaling pathway, reprograms immunosuppressive TAMs into the proinflammatory M1‐like phenotype, and thereby enhances the activation of CD8^+^ T cells and NK cells, ultimately inhibiting tumors such as melanoma, colorectal cancer, and lung cancer [389].

### 7.2 Neurodegenerative Diseases

In neurodegenerative diseases, α‐Syn, a protein closely associated with PD, exacerbates the pathological process of PD by being released through MVBs and spreading between neurons [199, 390]. For Tau proteins and Aβ that potentially lead to AD, secretory autophagy is involved in the expulsion of Aβ aggregates, and as UcPS is enhanced, their extracellular diffusion is also intensified [391]. In contrast, Tau proteins are secreted via unconventional pathways and are regulated by proteins such as Rab7A, VAMP8, Syntaxin 6, and Syntaxin 8 [160, 392, 393]. In familial amyotrophic lateral sclerosis, mutated Sod1 misfolds in neuronal cells through mechanisms dependent on exosomes and independent of exosomes [394, 395, 396]. The loss of UcPS mediated by Plekhg5 leads to an accumulation of Sod1 within cells; while it delays microglial activation, it aggravates intrinsic cellular damage [397]. In Huntington's disease, the secretion of mutant Huntingtin protein (Htt‐Q74) relies on GRASP55 and autophagic pathways [398]. Moreover, the delivery of the FGF2 gene enhances the phagocytosis of fibrillar Aβ in the hippocampus and neurogenesis from neural stem cells to restore hippocampal function. This approach is closely related to various neurodegenerative diseases, including AD, PD, multiple sclerosis, and traumatic brain injury, and is considered a potential therapeutic target [139].

### 7.3 Infection and Immune Evasion

Regarding inflammation and immune‐related diseases, in psoriasis, HMGB1 can activate dermal γδT cells to produce IL‐17A, leading to skin inflammation. After keratinocytes are activated through the MAPK pathway, they induce autophagy and secrete the critical protein HMGB1 via an unconventional secretion pathway that depends on ATG5 and GORASP2. This amplifies the immune cycle and exacerbates skin inflammation. Targeted inhibition of this process can effectively alleviate inflammatory responses [399]. In systemic lupus erythematosus, opsonized red blood cells that retain mitochondria (Mito^+^ RBCs) release mitochondrial RNA, which activates the RIG‐I‐like receptors–MAVS pathway in monocytes. This activation promotes the release of the monocytes’ own mitochondrial DNA and activates the NLRP3 inflammasome, and the activated Caspase‐1 cleaves pro‐IL‐1β into mIL‐1β. Subsequently, the myxovirus‐resistant protein 1 induced by IFN forms a functional structure through oligomerization, associates with the membrane of the TGN, and mediates the transmembrane transport of mIL‐1β into the TGN lumen. Finally, mIL‐1β is released outside the cell relying on the TGN‐mediated secretory mechanism, and this secretion process is independent of GSDMD and pyroptosis [400]. In rheumatoid arthritis, the UcPS of proinflammatory factors such as IL‐1β, along with dendritic cells presenting citrullinated antigens via major histocompatibility complex class II, jointly drive joint inflammation, autoimmune reactions, and bone destruction [401]. The envelope (E) proteins of severe coronaviruses (SARS‐CoV‐2, SARS, and MERS) bind to the host protein TMED10 via their unique SS/DS motif, promoting the oligomerization of TMED10. This interaction thereby activates the THU pathway, accelerating the translocation and release of inflammatory factors such as IL1β and IL33 into the ERGIC, and ultimately exacerbating lung inflammation [402]. The secretion of IL‐33 protein can also occur via the THU pathway. In astrocytes, under specific stimulation by IFN‐γ, intranuclear IL‐33 undergoes lysine acetylation modification to achieve nucleocytoplasmic transport. Subsequently, it binds to TMED10 via its own IL‐1‐like cytokine domain and is then transported to the extracellular space, thereby regulating experimental autoimmune encephalomyelitis and CNS homeostasis‐related diseases [403]. A similar secretion process occurs in intestinal epithelial cells: the GOLD domain and CT domain of TMED10 mediate the entry of IL‐33 into the ERGIC to initiate secretion, thereby influencing colitis [404].

For HIV, UcPS has also shown its therapeutic potential. Targeting the UcPS of HIV‐Tat protein holds the potential for synergistic effects on controlling multisystem complications of HIV. In HAND, Tat activates the calpain‐1/p25/CDK5 signaling axis through calcium homeostasis imbalance, leading to abnormal phosphorylation of neuronal cytoskeletal proteins like Tau and CRMP2, resulting in synaptic degeneration and apoptosis [140]. In HIV‐associated nephropathy, Tat collaborates with APOL1 risk variants to upregulate proinflammatory cytokines (IL‐6/TNF‐α), exacerbating podocyte injury and glomerular sclerosis [141]. By disrupting mitochondrial calcium homeostasis and autophagic clearance in cardiomyocytes, it induces contractile/diastolic dysfunction and mitochondrial damage. Through the NF‐κB pathway, it activates vascular endothelial inflammatory factors (IL‐1β, VCAM‐1) and adhesion molecules (ICAM‐1), accelerating atherosclerosis progression [142]. Blocking Tat secretion not only suppresses viral replication but also provides precise therapeutic targets by modulating CDK5 nucleocytoplasmic shuttling, APOL1 expression, and multiple channel interactions, beneficial for treating neurodegenerative diseases, organ failure, and complications in HIV‐infected individuals.

### 7.4 Therapeutic Strategies Targeting Secretory Mechanisms

Based on the previously discussed driving roles of UcPS in pathological processes, intervening at secretory nodes has emerged as a pivotal direction for precision medicine. Current strategies extend beyond merely clearing pathogenic proteins; they also leverage UcPS mechanisms for the delivery of therapeutic cargoes, resulting in a multidimensional intervention system encompassing engineered exosomes, small‐molecule inhibitors, and autophagy modulators [405].

Exosomes, serving as natural nanocarriers that mediate intercellular molecular communication, possess inherent bioactivity but frequently encounter bottlenecks in clinical translation, primarily due to low loading efficiency and insufficient targeting precision. To circumvent these limitations, researchers have leveraged genetic engineering to reprogram donor cells, endowing engineered exosomes with extended systemic circulation half‐lives and enhanced blood–brain barrier permeability [405]. In oncological therapy, engineered exosomes have shown considerable promise; for instance, exosomes displaying PD‐L1 inhibitors can effectively reverse the immunosuppressive microenvironment in breast cancer, resulting in significant tumor growth inhibition in murine models [406]. For CNS disorders, rabies virus glycoprotein‐modified exosomes facilitate the targeted delivery of siRNA or protein inhibitors to neurons, thereby reducing pathological protein aggregation and alleviating neuroinflammation, though delivery efficiency and long‐term safety remain formidable hurdles for clinical application [405]. Regarding systemic diseases and inflammatory regulation, the inhaled stem cell exosome paradigm demonstrates exceptional translational potential, as it enables localized mitigation of respiratory damage via pulmonary administration followed by systemic entry [407]; moreover, these vesicles can target the injured myocardium to activate cardiomyocyte proliferation and promote microvascular angiogenesis, facilitating functional remodeling after myocardial infarction [408]. Furthermore, molecules such as miR‐199a‐3p carried by exosomes have been shown to modulate inflammatory responses in kidney protection and sepsis models, offering novel intervention strategies for critical care medicine [409].

Beyond employing vesicles as delivery vehicles, the direct intervention of key molecular components within secretory pathways represents a critical strategy for intercepting pathological signaling. Taking tumor multidrug resistance as an example, the overexpression of ABC transporters is a central mechanism. The Harperoids series of compounds can inhibit their efflux function, thereby restoring chemosensitivity at the cellular level [410]. In the field of inflammation, disulfiram mitigates cytokine storms in murine sepsis models by covalently modifying the Cys191/192 residues of GSDMD to block pyroptotic pore formation [411]. This paradigm is currently being extended to neurodegenerative diseases. Mutations in VPS35 (e.g., D620N) lead to endosomal sorting defects, promoting PD‐associated protein aggregation. While restoring VPS35 function has been shown to reduce pathological deposition in cellular models [412], CNS delivery and long‐term safety remain formidable challenges.

At a deeper cellular level, secretory autophagy—the export of intracellular materials via unconventional routes—exhibits a dual effect: it serves both to clear damaged proteins to alleviate metabolic stress and as a conduit for the trans‐cellular propagation of pathogenic factors. Consequently, therapeutic interventions must be meticulously tailored to the specific pathological context rather than relying on binary activation or inhibition. In neurodegenerative diseases, for instance, Sec autophagy is involved in the transport of pathogenic agents such as α‐Syn and IL‐1β. Modulating the carrier fusion mediated by the SNARE complex can directly influence the dissemination of pathological signals [413]. Cancer therapy presents an alternative logic. TRIM16, as a context‐dependent autophagy regulator, can reshape the tumor immune microenvironment and modulate oxidative stress [414]. However, the role of TRIM16 is highly heterogeneous, exerting either tumor‐suppressive or prosurvival effects depending on the biological context; distinguishing these specific scenarios is a prerequisite for its pharmacological development. Ocular degenerative disorders shift the focus toward intervening in the secretion of the retinal pigment epithelium. For age‐related macular degeneration, modulating secretory autophagy to reduce the accumulation of drusen can delay retinal functional decline [415]. However, as extracellular deposits serve dual roles as both pathological byproducts and carriers of metabolic waste, excessive inhibition may inadvertently increase cellular burden. Overall, the regulation of secretory autophagy is transitioning from conceptualization to validation, and defining the boundary between “physiological defense” and “pathological diffusion” remains a critical hurdle for clinical translation.

Therapeutic strategies targeting secretory abnormalities are undergoing a transition from localized intervention to systemic regulation. Despite preliminary progress in vehicle‐based delivery and signaling blockade, clinical translation remains constrained by several key bottlenecks: suboptimal in vivo targeting efficiency of carriers, complex compensatory mechanisms between signaling pathways, and the difficulty of balancing exogenous intervention with endogenous homeostasis [416, 417]. In the future, the construction of intelligent systems capable of dynamically sensing lesion signals and achieving precision regulation is expected to drive a paradigm shift from “broad‐spectrum inhibition” toward “microenvironment‐responsive” therapy, opening new avenues for the intervention of complex diseases.

## 8 Conclusion and Future Perspectives

This review systematically summarizes the diverse mechanisms of UcPS in eukaryotes and prokaryotes, as well as their critical roles in physiology and disease. In eukaryotes, UcPS enables the rapid release of proteins under stress or pathological conditions through direct transmembrane transport, vesicle‐mediated secretion (e.g., exosomes, MVs, and autophagosomes), and Golgi‐bypass pathways, thereby overcoming the limitations of the conventional ER–Golgi pathway. Meanwhile, prokaryotes have evolved sophisticated nanomachines, ranging from the highly conserved Sec and Tat pathways to specialized T1SS–T11SS, to precisely deliver effector proteins and manipulate the host environment. Although eukaryotic UcPS and prokaryotic‐specific secretion systems appear distinctly different, we propose that they primarily aim to address an inherently similar problem: how organisms rapidly transport specific proteins across one or multiple lipid bilayers under particular stress states. Despite the structural differences between the eukaryotic endomembrane system and the bacterial envelope, these pathways adhere to several common design principles in the molecular modules for protein secretion.

First, both eukaryotes and prokaryotes have evolved one‐step, signal peptide‐independent secretion pathways for effector proteins. In eukaryotic Type I/II UcPS, cytosolic proteins such as FGF2, HIV‐Tat, and IL‐1β directly traverse the plasma membrane via self‐assembled lipidic pores or inflammasome‐driven heterologous pores. In bacteria, T1SS, T3SS, T6SS, and T7SS also bypass intermediate compartments, utilizing ABC transporters or contractile needle‐like nanomachines to translocate effector proteins from the cytoplasm to the extracellular environment or directly into target cells. For both secretion modes, the core challenge lies in achieving selectivity in the absence of classical N‐terminal signal peptides: eukaryotic UcPS relies on environment‐dependent motifs, charge‐dependent interactions between proteins and lipid membranes, and various stress sensors, while bacterial systems employ specialized molecular chaperones, C‐terminal secretion signals, and SPs. Second, pore formation is a common solution for transmembrane translocation. Eukaryotic cells utilize transient protein‐lipid assemblies (FGF2 oligomers, Tat oligomers) or proteolytically activated pore‐forming proteins (GSDMD) to pierce membranes and release cargo. Similarly, T3SS needle structures and T6SS phage tail‐like spikes physically penetrate the membranes of host or competitor cells to deliver effectors. Despite structural differences, the functional outcomes are similar: rapid, localized barrier penetration to enable acute responses such as cytokine release or toxin delivery. Furthermore, a notable evolutionary link is the ABC transporters. ABC transporters drive bacterial T1SS and also participate in certain eukaryotic Type II UcPS, indicating that ATP‐powered translocation mechanisms have been conserved during evolution and repurposed for different substrates and contexts.

Despite significant progress in research, there are still contradictions and limitations worthy of critical reflection in this field. First, there is inadequate depth in the elucidation of the mechanism. The molecular regulatory network of UcPS contains numerous unknown elements. For example, it is unclear whether these proteins follow unconventional pathways due to the action of sequences similar to signal peptides. It remains to be determined whether a single protein cargo utilizes multiple unconventional secretion pathways simultaneously (such as whether IL‐1β simultaneously undergoes GSDMD pore‐mediated leakage, exosomal secretion, and secretory autophagy). Whether there is a dynamic switching mechanism between different secretion pathways is also unknown (for instance, autophagosomes can fuse with MVBs for release; whether this can associate secretory autophagy with exosome release). Additionally, how different conformations of protein cargo affect their secretion methods, as well as the precise regulatory logic behind the formation and closure of membrane pores, all await further exploration. Second, it is difficult for in vitro experiments to simulate the dynamic changes of UcPS in the complex microenvironment within the body (such as multicellular interactions in tumor microenvironments), leading to a gap between mechanism research and clinical translation that is hard to bridge due to limitations in technical approaches. Under pathological conditions, UcPS often exhibits synergistic actions through multiple pathways. Therefore, how to precisely regulate specific disease‐related UcPS pathways without affecting normal physiological functions (such as inhibiting the release of tumor exosomes without disrupting immune cell communication) also needs to be urgently addressed. Thirdly, the protein components in peripheral blood, cerebrospinal fluid, and cellular microenvironments are complex and variable. This indicates that a large number of proteins are secreted via nonclassical Sec pathways, rather than being released into the extracellular space through cell rupture as previously hypothesized. Notably, the unexpected secretion of certain proteins and posttranslational modification substrates provides new clues for their functions, such as PTEN [389], TFRC [219], and ISG15 [418], whose secretory mechanisms remain unclear. It is unknown whether they are secreted through one of the aforementioned pathways or if they are modified first, secreted extracellularly alongside other proteins, and then detached. Additionally, whether the latter scenario constitutes a unique secretory mode warrants further investigation. Finally, secretory autophagy has garnered increasing attention; however, current research remains fragmented, making it difficult to sort out clear regulatory pathways. In particular, it is unclear whether autophagosomes enter the Sec autophagy pathway passively due to impaired autolysosome formation or via an unknown active selection mechanism. Recent studies have shown that some autophagosomal components can be recycled from lysosomes, and whether this process gives rise to a new autophagic secretion mode requires further exploration.

Technological innovation is accelerating research on UcPS and prokaryotic secretion systems. First, the widespread use of high‐resolution structural and dynamic imaging technologies, such as cryo‐electron microscopy and single‐molecule fluorescence imaging, facilitates the exploration of long‐standing ambiguous issues in aspects like the transient assembly states of secretion nanomachines, pore formation, and vesicle fusion. Additionally, quantitative and highly specific identification technologies are also areas with good development. Methods such as the combination of the sensitivity of split NanoLuc Binary Technology and the versatility of the retention using selective hooks system [419], and quantitative LC–MS/MS‐based secretomics [420], can not only rapidly detect low‐abundance unconventional secreted proteins but also locate the intermediate compartments of UcPS cargo, overcoming the limitations of traditional Western blot experiments.

Research on UcPS holds immense promise for updating our overall understanding of cell communication and protein homeostasis, providing a robust theoretical framework for targeted interventions. Therapeutic strategies are already transitioning from “broad‐spectrum inhibition” toward “microenvironment‐responsive” therapies. For example, engineered exosomes are being reprogrammed to overcome low loading efficiency and poor targeting, allowing them to cross the blood–brain barrier for neurodegenerative therapy or to deliver PD‐L1 inhibitors directly to tumor microenvironments. Additionally, direct pharmacological intervention at critical secetory nodes, such as using disulfiram to block GSDMD pyroptotic pore formation in sepsis, or harperoids to reverse ABC transporter‐mediated multidrug resistance, demonstrates high translational viability. Ultimately, resolving the remaining unknowns in UcPS regulation will not only address long‐standing questions in fundamental cell biology but also bridge the gap between molecular mechanisms and clinical applications, cementing UcPS as a central focal point in future translational medicine.

## Author Contributions

Q.Y. and J.S. wrote the main part of the manuscript. L.X., G.Z., and Y.C. wrote several parts of the manuscript. L.C., X.L., Z.S., and S.H. made a substantive revision of the text, added essential paragraphs and conclusions, and wrote the final version of the manuscript. The manuscript has been read and approved by all coauthors. All the authors corrected and approved the final version of the manuscript.

## Funding

X.L. was supported by the Shandong Provincial Natural Science Foundation (number: ZR2023QH459), and S.H. was supported by the Shandong Provincial Natural Science Foundation (numbers: ZR2024MH138 and ZR2022LZL006).

## Conflicts of Interest

The authors declare no conflicts of interest.

## Ethics Statement

The authors have nothing to report.

## Data Availability

The authors have nothing to report.
